# Effects of climate extremes on the terrestrial carbon cycle: concepts, processes and potential future impacts

**DOI:** 10.1111/gcb.12916

**Published:** 2015-05-12

**Authors:** Dorothea Frank, Markus Reichstein, Michael Bahn, Kirsten Thonicke, David Frank, Miguel D Mahecha, Pete Smith, Marijn van der Velde, Sara Vicca, Flurin Babst, Christian Beer, Nina Buchmann, Josep G Canadell, Philippe Ciais, Wolfgang Cramer, Andreas Ibrom, Franco Miglietta, Ben Poulter, Anja Rammig, Sonia I Seneviratne, Ariane Walz, Martin Wattenbach, Miguel A Zavala, Jakob Zscheischler

**Affiliations:** 1 Max Planck Institute for Biogeochemistry07745, Jena, Germany; 2 Institute of Ecology, University of Innsbruck6020, Innsbruck, Austria; 3 Potsdam Institute for Climate Impact Research (PIK) e.V.14773, Potsdam, Germany; 4 Berlin-Brandenburg Institute of Advanced Biodiversity Research (BBIB)14195, Berlin, Germany; 5 Swiss Federal Research Institute WSL8903, Birmensdorf, Switzerland; 6 Oeschger Centre for Climate Change Research, University of BernCH-3012, Bern, Switzerland; 7 Institute of Biological and Environmental Sciences, University of Aberdeen23 St Machar Drive, Aberdeen, AB24 3UU, UK; 8 Ecosystems Services and Management Program, International Institute of Applied Systems Analysis (IIASA)A-2361, Laxenburg, Austria; 9 Research Group of Plant and Vegetation Ecology, Biology Department, University of AntwerpWilrijk, Belgium; 10 Laboratory of Tree-Ring Research, The University of Arizona1215 E Lowell St, Tucson, AZ, 85721, USA; 11 Department of Environmental Science and Analytical Chemistry (ACES), Bolin Centre for Climate Research, Stockholm University10691, Stockholm, Sweden; 12 ETH Zurich8092, Zurich, Switzerland; 13 Global Carbon Project, CSIRO Oceans and Atmosphere FlagshipGPO Box 3023, Canberra, ACT, 2601, Australia; 14 IPSL – Laboratoire des Sciences du Climat et de l’Environnement CEA-CNRS-UVSQ91191, Gif sur Yvette, France; 15 Institut Méditerranéen de Biodiversité et d’Ecologie marine et continentale (IMBE), Aix Marseille Université, CNRS, IRD, Avignon UniversitéAix-en-Provence, France; 16 Department of Chemical and Biochemical Engineering, Technical University of Denmark (DTU)Frederiksborgvej 399, 4000, Roskilde, Denmark; 17 IBIMET-CNRVia Caproni, 8, 50145, Firenze, Italy; 18 FoxLab, Fondazione E.MachVia Mach 1, 30158, San Michele a/Adige, Trento, Italy; 19 Institute of Earth and Environmental Science, University of Potsdam14476, Potsdam, Germany; 20 Helmholtz Centre Potsdam, GFZ German Research Centre For Geosciences14473, Potsdam, Germany; 21 Forest Ecology and Restoration Group, Universidad de AlcaláAlcalá de Henares, Madrid, Spain

**Keywords:** carbon cycle, climate change, climate extremes, climate variability, disturbance, terrestrial ecosystems

## Abstract

Extreme droughts, heat waves, frosts, precipitation, wind storms and other climate extremes may impact the structure, composition and functioning of terrestrial ecosystems, and thus carbon cycling and its feedbacks to the climate system. Yet, the interconnected avenues through which climate extremes drive ecological and physiological processes and alter the carbon balance are poorly understood. Here, we review the literature on carbon cycle relevant responses of ecosystems to extreme climatic events. Given that impacts of climate extremes are considered disturbances, we assume the respective general disturbance-induced mechanisms and processes to also operate in an extreme context. The paucity of well-defined studies currently renders a quantitative meta-analysis impossible, but permits us to develop a deductive framework for identifying the main mechanisms (and coupling thereof) through which climate extremes may act on the carbon cycle. We find that ecosystem responses can exceed the duration of the climate impacts via lagged effects on the carbon cycle. The expected regional impacts of future climate extremes will depend on changes in the probability and severity of their occurrence, on the compound effects and timing of different climate extremes, and on the vulnerability of each land-cover type modulated by management. Although processes and sensitivities differ among biomes, based on expert opinion, we expect forests to exhibit the largest net effect of extremes due to their large carbon pools and fluxes, potentially large indirect and lagged impacts, and long recovery time to regain previous stocks. At the global scale, we presume that droughts have the strongest and most widespread effects on terrestrial carbon cycling. Comparing impacts of climate extremes identified via remote sensing vs. ground-based observational case studies reveals that many regions in the (sub-)tropics are understudied. Hence, regional investigations are needed to allow a global upscaling of the impacts of climate extremes on global carbon–climate feedbacks.

## Introduction

There is widespread recognition that climate change is having and will continue to have, fundamental impacts on the natural environment and on human well-being (Parry *et al*., 2007). Current projections, based upon contrasted emission scenarios, suggest somewhere between 0.3 and 4.8 °C warming by the end of this century (IPCC, 2013). The associated modification of the climate system strongly influences the carbon cycling of the terrestrial biosphere and thus land–atmosphere CO_2_ fluxes (Fischlin *et al*., 2007). An important observation is that climate change, and increasing concentrations of atmospheric greenhouse gases, not only lead to gradual mean global warming but may also change the frequency, the severity and even the nature of extreme events (IPCC, 2013). A relatively small change in the mean or variance of a climate variable, inherently leads to disproportionally large changes in the frequency of extremes, that is the infrequent events at the high and low end of the range of values of a particular variable (Nicholls & Alexander, 2007). Furthermore, climate change can fundamentally alter the inherent variability of temperature, precipitation and other weather phenomena (Seneviratne *et al*., 2012). State-of-the-art climate models project global intensification of heavy precipitation events and heat extremes, and regions with stronger or longer-lasting droughts (Fisher & Knutti, 2014, IPCC, 2013).

Concerns about increasing variability of temperature and precipitation patterns and climate extremes were first articulated over two decades ago by Katz & Brown (1992), and became widely acknowledged after the second IPCC assessment of climate change in 1995 (Nicholls & Alexander, 2007). These concerns were raised because many biological systems (including human societies) are more sensitive to climate extremes than to gradual climate change, due to typically greater response strengths and shorter response times (Hanson *et al*., 2006).

Key characteristics of the climate, such as heat waves, seem to have already been modified beyond the natural variability within which society and its economic, social and political systems have developed (Schär *et al*., 2004; Soussana *et al*., 2010). Both the public media and the scientific community have recognized the widespread consequences of climate extremes such as the European heat wave in 2003 (Ciais *et al*., 2005; Reichstein *et al*., 2007; Bastos *et al*., 2013a), the heat wave and associated forest fires in Greece in 2007 (Founda & Giannakopoulos, 2009), the dry spells in the Amazon basin in 2005 (Phillips *et al*., 2009) and 2010 (Lewis *et al*., 2011), in the U.S.A. 2000–2004 (Breshears *et al*., 2005; Schwalm *et al*., 2012), the forest fires in Russia in 2010 (Barriopedro *et al*., 2011; Konovalov *et al*., 2011; Coumou & Rahmstorf, 2012; Bastos *et al*., 2013a), the Pakistan Floods in 2010 (Hong *et al*., 2011; Houze *et al*., 2011; Trenberth & Fasullo, 2012), the storm Lothar in Europe in 1999 (Lindroth *et al*., 2009), hurricane Katrina in the U.S. in 2005 (Chambers *et al*., 2007), or the ice storm in southern and central China in 2008 (Stone, 2008; Sun *et al*., 2012), and the 2010–2011 La Nina rains over Australia (Boening *et al*., 2012; Poulter *et al*., 2014). These documented recent events demonstrate the massive impacts climate extremes can have on harvests, economies and human health, as well as on the carbon balance of terrestrial ecosystems (IPCC, 2012; Reichstein *et al*., 2013).

Alterations of the biosphere’s carbon balance through changes in the strength of carbon uptake or losses in turn affect the climate system (Friedlingstein *et al.,* 2006; Frank *et al.,* 2010). In addition, extreme drought will often reduce evapotranspiration and its cooling effect and thereby causes a positive local feedback on warming (e.g. Seneviratne *et al*., 2010; Teuling *et al*., 2010; Mueller & Seneviratne, 2012; Peng *et al*., 2014). Regional assessments clearly indicate the relevance of climate extremes on the carbon cycle and potential climate feedbacks (e.g. for drought extreme in Europe, Ciais *et al*., 2005; Reichstein *et al*., 2007; and for western North America, Schwalm *et al*., 2012). Yet a synthesis of the direct and indirect impacts of climate extremes on the carbon cycle and the underlying mechanisms is still lacking. In a recent broad perspective, Reichstein *et al*. (2013) highlighted the possibility that climate extremes and their impacts on the global carbon cycle may lead to an amplification of positive climate–carbon cycle feedbacks. However, the underlying mechanisms, and how they likely apply to current and future response patterns observed in different biomes and ecosystem types, have not yet been synthesized in detail, especially with respect to possible differences in response time (concurrent/lagged) and direction of impacts (direct/indirect). Such detailed information is needed, given the complexity of carbon cycle responses to climate extremes, and their dependence on background climate and ecosystem conditions (Knapp *et al*., 2008).

In this review, we aim to (1) develop a coherent conceptual framework based on logically deductive reasoning for integrating direct and indirect effects climate extremes could have on the carbon cycle and to identify the main mechanisms underlying these effects, (2) synthesize how different types of ecosystems are affected by climate extremes based on available well-documented case studies and (3) provide an overview of likely responses of the terrestrial carbon cycle in relation to likely future climate extremes, and the specific role of lagged impacts.

At the outset, we acknowledge that the lack of systematically collected data and the highly nonlinear responses of ecosystems to extreme events makes a quantitative meta-analysis of effects of climate extremes on the carbon cycle across the range of observational and experimental studies virtually impossible (cf. also Vicca *et al*., 2014). While there is ample information in the literature on specific effects of extreme climatic conditions (experimentally induced or naturally occurring) on specific ecosystems, the severity of these extreme conditions and their consequences has often not been systematically evaluated. This is not only due to a lack of common metrics reported across the various studies (e.g. Vicca *et al*., 2012), but also complicated by the fact that climate extremes are by definition rare and their effects are highly context dependent, typically threshold based and highly nonlinear (e.g. Knapp *et al*., 2008; Smith, 2011, Bahn *et al*., 2014). Thus, in our review, we rely on a qualitative, logically deductive reasoning, supported by multiple case studies, combined with remote sensing-based global analysis to derive hypotheses on potential effects of climate extremes on the terrestrial carbon cycle.

## Definitions

### Climate extremes and impacts

Terms, such as ‘climate extremes’, ‘weather extremes’ or ‘extreme weather events’, are used in various ways in the scientific literature. Thus, for clarity, we provide and briefly justify the definitions we use in this review:

An ‘extreme’, as stated in Seneviratne *et al*. (2012), is ‘the occurrence of a value of a weather or climate variable above (or below) a threshold value near the upper (or lower) ends of the range of observed values of the variable’ within a defined climate reference period (e.g. 1981–2010). Thus, ‘climate extreme’ is an aggregate term encompassing both ‘extreme weather’ and ‘extreme climate’ events. The distinction of weather events and climate events is related to the timescale. An extreme climate event occurs on longer timescales than an extreme weather event and can be the accumulation of extreme weather events. This definition follows the IPCC Special Report on Managing the Risks of Extreme Events and Disasters to Advance Climate Change Adaptation (Seneviratne *et al*., 2012).

However, the above definitions reflecting climatological considerations do not consider potential consequences for the biosphere and the carbon cycle. Smith (2011) suggested that one has to specifically address events where both climates are anomalous and the biosphere experiences a pronounced impact outside the bounds of what is considered normal variability. Along these lines, we use the term ‘extreme impact’ to describe, from a functional perspective, when a resilience threshold (‘extreme response threshold’, *sensu* Smith, 2011) is passed, placing the ecosystem and associated carbon cycling into an unusual or rare state. Thresholds are typically exceeded when stressor dose (i.e. cumulative amount defined by stress intensity multiplied by stress duration) reaches a critical level (e.g. during flooding, drought and/or extended periods of exceptionally high or low temperatures), or when the intensity of an extreme climatic event is critically high (e.g. during a storm). Thresholds can be passed at organ, plant or community level, and lead to emergent carbon cycle impacts at ecosystem level. We note that the definition of ‘extreme impact’ may partly overlap with the concept of ‘disturbance’ as it is commonly used in ecology (White & Jentsch, 2001). Here, we consider every climate extreme which has an impact on the ecosystem carbon cycle a ‘disturbance’, but note that not every disturbance is caused by climate extremes. A typical example is fire, which can be part of a system intrinsic disturbance cycle. But in this study, we consider those fires which are of rare magnitude or even are unprecedented, and likely facilitated by extreme climate conditions. Given that impacts caused by ‘climate extremes’ can be considered ‘disturbances’, we assume that respective general mechanisms and processes induced by ‘disturbances’ also operate in this specific ‘extreme’ context.

**Figure 1:**
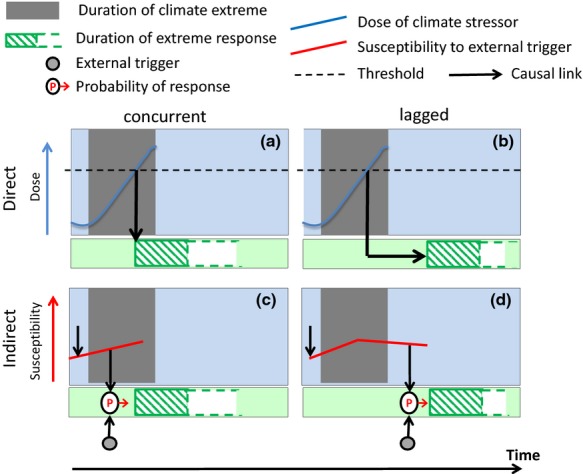
Schematic diagram illustrating direct concurrent and lagged (a, b) and indirect concurrent and lagged (c, d) impacts of climate extremes and corresponding extreme ecosystem responses. In the direct case, the extreme impact occurs if (and only if) a threshold is reached, that is a critical dose (blue line) is passed. In the indirect case, the climate extreme increases the susceptibility (red line) to an external trigger (climatic or nonclimatic, extreme or not extreme). The likelihood as a function of the trigger and the susceptibility is indicated with the symbol ‘P’ in the circle. Concurrent responses start during the climate extreme, but may last longer for indefinite time (dashed extensions of green boxes). Lagged responses only happen after the climate extreme. The responses can be of different nonlinear shapes as indicated in Fig.2.

In order to specifically address extreme impacts with repercussions to the carbon cycle, denoted as ‘carbon cycle extreme’ and to entail anomalies in biosphere–atmosphere carbon fluxes or extreme changes in ecosystem carbon pools, it is useful to distinguish ‘concurrent’ vs. ‘lagged’ and ‘direct’ vs. ‘indirect’ impacts (Fig.1). These four categories of impacts indicate how they are related to the stressor. Concurrent impacts begin to occur during the climate extreme, while lagged impacts occur sometime thereafter. Direct impacts are only caused by the climate extreme (either concurrently or lagged) if, and only if, a threshold of the climatic stress dose (dashed line in Fig.1a) is passed. Indirect impacts are facilitated by the climate extreme by increasing the susceptibility of the ecosystem, but directly initiated by another (not necessarily extreme *per se*) external trigger. Hence, here the likelihood (P) of an extreme system response is a function of both the susceptibility and the characteristics of the external trigger (cf. Fig.1b and d).

Examples for these four categories of impacts are as follows:

1. Direct, concurrent impact: windthrow caused by storm; ice breakage; reduced productivity or increased mortality during drought, thermal stress or flooding (cf. Fig.1a)
2. Indirect, concurrent impact: loss of biomass or soil organic matter due to fire caused by lightning or human ignition, facilitated by an ongoing extreme dry and/or warm event (cf. Fig.1b)
3. Direct, lagged impact: reduced productivity/growth in the year(s) following the year of an extreme drought, caused, for example by carbohydrate depletion/reduced bud development/partial mortality during a drought in the previous year (cf. Fig.1c)
4. Indirect, lagged impact: increased pest- or pathogen-caused mortality following a climate extreme; loss of biomass or soil organic matter due to fire facilitated through deadwood accumulation after a windthrow; loss of soil carbon due to erosion during heavy precipitation or permafrost thawing and carbon losses as indirectly facilitated by reduced vegetation cover and/or changes in soil hydrophobicity following overgrazing, drought or fire (cf. Fig.1d)

Any effect, which can be attributed to a previous climate extreme, is termed here a ‘legacy effect’ and hence per definition time lagged compared to the ‘climate extreme’ [please note that we prefer this terminology compared to the sometimes synonymously used anthropomorphic term ‘memory effect’ (Walter *et al*., 2013)]. Legacy effects can include both changes in ecosystem states or process rates after the termination of a climate extreme, as well as altered ecosystem responses to environmental conditions, including subsequent extremes, and are often related to changes in species composition and their functional attributes (e.g. Smith, 2011; Sala *et al*., 2012).

**Figure 2:**
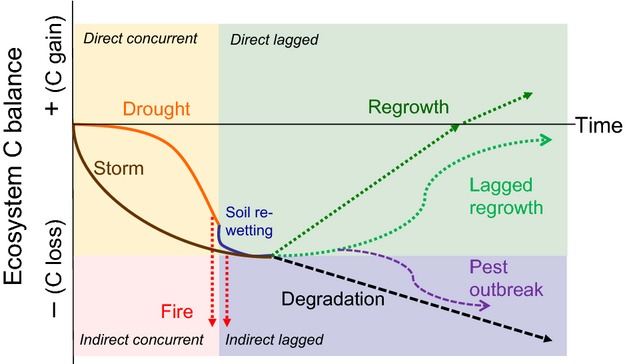
Hypothesized temporal dynamics of direct and indirect concurrent and lagged effects of climate extremes (e.g. drought/heat wave; storm) and of ecosystem recovery on the ecosystem carbon balance. (Note that for simplicity regrowth after fire and pest outbreaks are not shown in this figure). Line colours correspond to the colour of the climate extreme in the figure.

It should be noted that is essential to define the timescale under scrutiny when quantifying the overall effect of a ‘climate extreme’ on the carbon cycle (Fig.2). It is the timescale determining the degree to which concurrent and lagged effects alter the carbon balance of an ecosystem. Negative concurrent effects, often related to the resistance of an ecosystem to an extreme event, may in the long run be balanced by enhanced regrowth during recovery (Fig.2), depending on the resilience of the system. Lagged effects may impair the ability of an ecosystem to recover from an extreme event and may thereby alter the ecosystem carbon balance over a given period (Fig.2).

## Impacts of climate extremes on the terrestrial carbon cycle: mechanisms and processes

Climate extremes can impact the structure, composition and functioning of terrestrial ecosystems and can thereby severely affect the regional carbon cycle, with the potential of causing a shift from a carbon sink towards a carbon source. During the ‘European 2003 heat wave’, which was an extreme drought event, Western European ecosystems were estimated to have lost an amount of CO_2_ comparable to that which had been absorbed from the atmosphere during the previous three to five years under normal weather conditions (Ciais *et al*., 2005; Reichstein *et al*., 2007; Vetter *et al*., 2008). Likewise, during the 2000–2004 drought, the strength of the western North American carbon sink declined substantially, with reductions ranging between 30 and 298 Tg C yr^-1^ (Schwalm *et al*., 2012). In 2004, heavy precipitation associated with Typhoon Mindulle led to a particulate organic carbon flux of 0.5 Mt over a 96-h period, with subsequent rapid burial of the terrestrial carbon in the ocean (Goldsmith *et al*., 2008). Also, extreme wind storms and cyclones can severely impact the regional carbon balance: In 1999 storm, Lothar reduced the European C sink by 16 Mt C, which corresponds to 30% of Europe’s net biome production (Lindroth *et al*., 2009) and, hurricane Katrina in 2005 destroyed an amount equivalent to 50–140% of the net annual U.S. C sink in forest trees (Chambers *et al*., 2007). Fires, pest and pathogen outbreaks are obviously not climate extremes, but can be facilitated by climate extremes. Extreme fire events release large quantities of carbon to the atmosphere. For example, in Indonesia, people had drained and deforested tropical wetlands which they then ignited to burn the debris awaiting the rain season to extinguish the fires, which failed due to the onset of the strong El-Niño Southern Oscillation in 1997/1998, which instead burnt the duff layers and vegetation releasing between 0.81 and 2.57 Gt C (Page *et al*., 2002). This amount was equivalent to the estimated annual release (van der Werf *et al*., 2010) and, together with the extreme fire events occurring in Siberia, produced a signal detected by atmospheric CO_2_ and CH_4_ monitoring stations (Simpson *et al*., 2006). Pest and pathogen outbreaks can have large impacts on forest carbon stocks and fluxes, and may impact the regional carbon cycle (Hicke *et al*., 2012), as was the case in a mountain pine beetle outbreak in British Columbia of unprecedented extent and severity, which converted the forest from a small net carbon sink to a large net carbon source (during and immediately after the outbreak) with an estimated cumulative regional impact of 270 Mt C for 2000–2020 (Kurz *et al*., 2008b).

**Figure 3:**
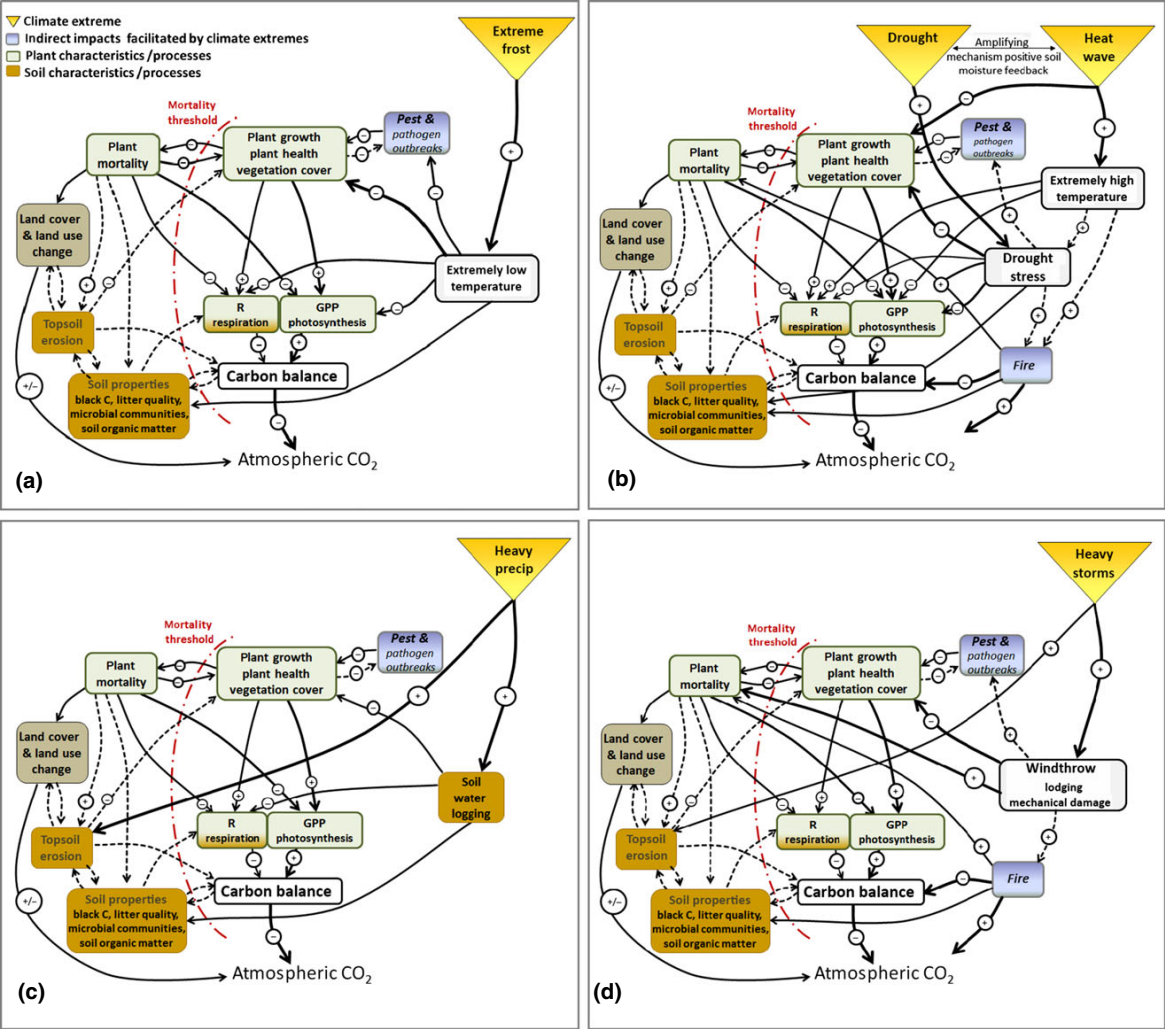
Processes and mechanisms underlying impacts of climate extremes on the carbon cycle. Positive/enhancing impacts with a ‘+’ and negative/reducing impacts with a ‘−’sign; predominant (in-)direct impacts (dashed) arrows (for further details please see text); importance of impact/relationship is shown by arrow width (high = thick, low = thin) (modified after Reichstein *et al*., 2013).

**Figure 4:**
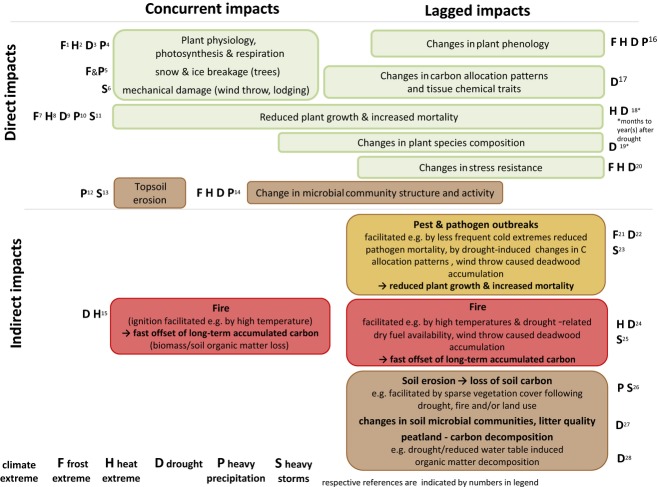
Schematic overview of concurrent, lagged, direct and indirect impacts of climate extremes on processes underlying ecosystem carbon dynamics. Respective references (selection of examples) are indicated as followed: ^1^ Larcher (2003) and Mayr *et al*. (2007); ^2^ Larcher (2003), Schulze *et al*. (2005), Lobell *et al*. (2012), Porter & Semenov (2005) and Niu *et al*. (2014); ^3^ Larcher (2003), Bréda *et al*. (2006), Keenan *et al*. (2010), Reichstein *et al*. (2007), Misson *et al*. (2010), Schwalm *et al*. (2010) and Eamus *et al*. (2013); ^4^ Rosenzweig *et al*. (2002), Vervuren *et al*. (2003), Kreuzwieser *et al*. (2004) and van der Velde *et al*. (2012); ^5^ Nykänen *et al*. (1997), Irland (2000), Changnon (2003), Hao *et al*. (2011) and Sun *et al*. (2012); ^6^ Berry *et al*. (2003), Fuhrer *et al*. (2006), MCPFE (2007), Lindroth *et al*. (2009), Zeng *et al*. (2009) and Negrón-Juárez *et al*. (2010b); ^7^ Larcher (2003), Schulze *et al*. (2005), Dittmar *et al*. (2006) and Bokhorst *et al*. (2009); ^8^ Larcher (2003), Porter & Semenov (2005), Bréda *et al*. (2006) and Lobell *et al*. (2012); ^9^ Barber *et al*. (2000), Eilmann *et al*. (2011), Fuhrer *et al*. (2006), Phillips *et al*. (2009), Michaelian *et al*. (2011), McDowell *et al*. (2013) and Peñuelas *et al*. (2013); ^10^ Vervuren *et al*. (2003) and Posthumus *et al*. (2009); ^11^ MCPFE (2007), Chambers *et al*. (2007), Zeng *et al*. (2009) and Negrón-Juárez *et al*. (2010a,b); ^12^ Fuhrer *et al*. (2006), Hilton *et al*. (2008) and García-Ruiz *et al*. (2013); ^13^ Wang *et al*. (2006) and Shinoda *et al*. (2011); ^14^ Jentsch *et al*. (2011) and Fuchslueger *et al*. (2014); ^15^ Moriondo *et al*. (2006) and Ganteaume *et al*. (2013); ^16^ Porter & Semenov (2005), Jentsch *et al*. (2009), Misson *et al*. (2011), Nagy *et al*. (2013) and Peñuelas *et al*. (2013); ^17^ Bréda *et al*. (2006), McDowell *et al*. (2008, 2011, 2013) and Walter *et al*. (2012); ^18^ Bréda *et al*. (2006), Adams *et al*. (2009), Allen *et al*. (2010), Michaelian *et al*. (2011), McDowell *et al*. (2008, 2011) and Granda *et al*. (2013); ^19^ Kreyling *et al*. (2011), Suarez & Kitzberger (2008) and Diez *et al*. (2012); ^20^ Larcher (2003) and Walter *et al*. (2013); ^21^ Virtanen *et al*. (1998), Stahl *et al*. (2006), Robinet & Roques (2010) and Kausrud *et al*. (2012); ^22^ Bréda *et al*. (2006), Desprez-Loustau *et al*. (2006), Rouault *et al*. (2006), MCPFE (2007), McDowell *et al*. (2008, 2011), Jactel *et al*. (2012), Keith *et al*. (2012), Kausrud *et al*. (2012) and Walter *et al*. (2012); ^23^ Schlyter *et al*. (2006), MCPFE (2007) and Komonen *et al*. (2011); ^24^ Trigo *et al*. (2006) and Wendler *et al*. (2011); ^25^ Kurz *et al*. (2008a); ^26^ Øygarden (2003), Valentin *et al*. (2008) and Thothong *et al*. (2011); ^27^ Sheik *et al*. (2011), Yuste *et al*. (2011) and, Fuchslueger *et al*. (2014); ^28^ Sowerby *et al*. (2008).

To be able to generalize and project presumable impacts of climate extremes on the carbon cycle, an understanding of the likely mechanisms and processes involved in extreme impacts is crucial. In this section, we review the primary environmental–biological processes according to their hypothesized relevance to different ecosystems, and the cascade of associated consequences. The complex pathways of how climate extremes may act on the major processes and components of the terrestrial CO_2_ balance are illustrated in Fig.3. We then provide a schematic overview of possible concurrent, lagged, direct and indirect impacts of climate extremes on processes underlying ecosystem carbon dynamics highlighting the importance of lagged impacts (Fig.4).

## Direct impacts

Temperature extremes can directly and concurrently impact photosynthesis and respiration (cf. Fig.3a and b). Effects differ between species, ecosystem types and biomes, and may change seasonally and even diurnally through hardening responses (Larcher, 2003). Concurrent direct impacts of extremely high temperatures range from disruptions in enzyme activity affecting photosynthesis and respiration, to changes in growth and development (Larcher, 2003; Schulze *et al*., 2005; Lobell *et al*., 2012; Niu *et al*., 2014). Likewise, extremely low temperatures impact physiological functions and developmental processes. Frost damage is perhaps the most important direct concurrent impact of cold climate extremes. In this context, timing is a crucial factor: in temperate ecosystems risk of plant damage is particularly high in spring when temperatures drop below freezing after an early warming event (Bokhorst *et al*., 2009; Migliavacca *et al*., 2009), or during cold outbreaks when autumn hardening is insufficient, or when a protective snow cover is absent during extreme frost. In addition to frost damage of needles, xylem embolism in response to freeze-thaw cycles frequently adds to the factors decreasing plant vitality (Fig.3a) (Sperry & Sullivan, 1992; Mayr *et al*., 2003, 2007).

Unusual warming events at the end of the winter season in temperate and boreal climates can induce plant activity too early, a phenomenon that has been called ‘false spring’ (e.g. Marino *et al*., 2011). Extreme warm late winters together with the general trend of average warming may lead to earlier onset of the seasonal plant development, unfulfilled chilling requirements, that is the exposure to cool temperatures that is required before dormancy can be broken. A general trend of earlier onset of greening has been observed at local scales, from phenological gardens across Europe and globally from remote sensing NDVI data (Myneni *et al*., 1997; Menzel *et al*., 2006; Pilegaard *et al*., 2011). If plants switch from dormancy to physiological activity earlier, they may become more susceptible to frost events with strong negative consequences, such as tissue mortality (Polle *et al*., 1996), increased tree crown transparency (Dittmar & Elling, 2007), and reduced tree growth (Dittmar *et al*., 2006) and plant performance (Kreyling, 2010).

Drought extremes may have manifold impacts on the carbon cycle *via* direct concurrent impacts (e.g. on plant physiology and soil microbial activity), direct lagged impacts (e.g. on the phenology of plants, reduced growth in the following year due to lower carbohydrate storage in the year of the drought, altered composition of plant species, soil microbial community structure and activity), as well as indirect lagged impacts, for example by drought-facilitated pest and pathogen outbreaks or fire ignition and spread (see Figs3 and 4). Effects of drought on gross ecosystem productivity are typically larger than for ecosystem respiration (Schwalm *et al*., 2010; cf. Fig.3b).

Drought stress occurs whether the water potential of an organism/tissue drops below a critical threshold. For example, in temperate and Mediterranean forest ecosystems, decreased transpiration, gross photosynthesis and respiration were observed when relative root extractable soil water dropped below 40% (Granier *et al*., 2007). High temperatures and low relative humidity (often expressed at the vapour pressure deficit) serve to increase evaporative demand, and drought stress of plants occurs when soil water supply can no longer meet the plant evaporative demand (e.g. Sperry, 2000). Plant available water is influenced by soil type and local surface and subsurface characteristics, such as the depth to the groundwater level or bedrock. The amount of water actually available to a plant depends strongly on the distribution of soil water across the profile in relation to root depth and type (Schachtschabel *et al*., 1992; Tolk, 2003; White, 2006; Vicca *et al*., 2012).

Droughts and extreme high temperatures (heat waves), both to be considered climate extremes in their own right, cannot be seen as independent phenomena as in many (transitional climate) regions droughts additionally are connected with high temperature extremes (Mueller & Seneviratne, 2012) (Fig.3b). The combination of high temperatures and droughts initiate a positive regional feedback mechanism (e.g. Durre *et al*., 2000; Seneviratne *et al*., 2006; Fischer *et al*., 2007; Vautard *et al*., 2007; Zampieri *et al*., 2009; Diffenbaugh & Ashfaq, 2010; Hirschi *et al*., 2011): the precipitation deficits and enhanced evaporative demand generally associated with warm spells (e.g. atmospheric blockings) triggers soil moisture deficit, thus suppressing evaporative cooling (Teuling *et al*., 2010) and leading to hotter and drier conditions if soil moisture becomes limiting for evapotranspiration (Seneviratne *et al*., 2010). Warmer temperatures additionally increase vapour pressure deficit, even without a concurrent reduction in rainfall, and this process alone causes extra drought stress (Williams *et al*., 2012). In addition, there are likely also nonlocal feedbacks between drought conditions and heat waves, for instance through the advection of dry air or the modification of regional-scale circulation patterns (e.g. Vautard *et al*., 2007; Haarsma *et al*., 2009).

Plants may respond to drought stress by structural or physiological adjustments such as decreased leaf area index, changes in the root–shoot ratio, or changes in osmolyte concentration (Larcher, 2003; Bréda *et al*., 2006). The ability of plants to extract water from deeper layers under soil moisture stress, up to some limit, has been reported (e.g. Nepstad *et al*., 1994; Canadell *et al*., 1996; Wan *et al*., 2002; Teuling *et al*., 2006). Drought decreases CO_2_ assimilation rates (according to our definitions, a direct concurrent impact) by reducing stomatal and mesophyll conductance, the activity and concentrations of photosynthetic enzymes (Lawlor, 1995; Chaves *et al*., 2009; Keenan *et al*., 2010) and reducing sink strength (Palacio *et al*., 2014). Generally, direct concurrent drought impacts are larger for plant photosynthesis than for respiration of plants (Atkin & Macherel, 2009) and ecosystems (Schwalm *et al*., 2010; Shi *et al*., 2014) (Fig.3b).

In addition to direct concurrent drought impacts like decreased carbon (and nutrient) assimilation (Fig.3b), drought may have lagged impacts on the carbon cycle *via* the re-allocation of existing stored reserves for repair, maintenance (including that of hydraulic integrity), growth and defence, as well as indirect lagged impacts (Fig.4) by increasing the ecosystems’ vulnerability to additional stressors such as pests and pathogens, or subsequent drought events (Bréda *et al*., 2006; Desprez-Loustau *et al*., 2006; McDowell *et al*., 2011; Sala *et al*., 2012; Keith *et al*., 2012).

Water stress has a direct, concurrent impact on microbial activity, which depends on the presence of water films for substrate diffusion and exo-enzyme activity (Davidson & Janssens, 2006), whereas indirect and lagged drought impacts on microbial activity may be initiated by various mechanisms such as a decreased input of labile carbon into the soil due to reduced plant productivity (Araus *et al*., 2002; Reddy *et al*., 2004), and altered soil nutrient retention and availability (Muhr *et al*., 2010; Bloor & Bardgett, 2012). Drought may also alter microbial community structure (Sheik *et al*., 2011) with consequences for carbon cycling (Fig.4; direct concurrent and (in-)direct lagged impact via changes in species composition) (Fuchslueger *et al*., 2014). In Mediterranean ecosystems, for example, fungi were less affected by drought than bacteria and controlled soil organic matter decomposition (Curiel-Yuste *et al*., 2011). While soil and ecosystem respiration are reduced by drought, rewetting by rainfall following drought can strongly stimulate soil CO_2_ emissions to levels substantially exceeding predrought (or control) rates, with immediate consequences for the carbon cycle (Fig.2, Jarvis *et al*., 2007; see also reviews by Borken & Matzner, 2009; Kim *et al*., 2012; Vicca *et al*., 2014). Different mechanisms act when drying–rewetting cycles become more pronounced. Among others, physical disruption of aggregates (Borken & Matzner, 2009), increased soil water repellency (Goebel *et al*., 2011) and altered nutrient retention (Borken & Matzner, 2009; Bloor & Bardgett, 2012) can be responsible for legacy effects on microbial activity and respiration, by modifying substrate and nutrient availability (indirect and lagged impact).

The magnitude of the impact on key ecosystem processes from an altered quantity, frequency or intensity of precipitation critically depends on the ecosystems’ (seasonally varying) baseline water limitation (Gerten *et al*., 2008). In addition to intensity and duration, the timing of droughts is a crucial factor due to the pronounced seasonal cycle of many ecosystems and land uses (Allard *et al*., 2008; Unger *et al*., 2009; Misson *et al*., 2010, 2011; De Boeck *et al*., 2011).

Extreme precipitation events may alter soil CO_2_ fluxes and CO_2_ uptake by plants during water logging phases (direct concurrent impacts on the carbon cycle), may lead to flooding-related tree mortality (Kramer *et al*., 2007) and may cause topsoil erosion (Fig.3c; see also below and Fig.4) with losses of particulate and dissolved organic carbon from terrestrial to riverine ecosystems (Hilton *et al*., 2008; Dinsmore *et al*., 2013). In more water-limited systems, longer intervals between rainfall events may increase the duration and severity of soil drought stress. In contrast, longer intervals between heavy rainfall events may reduce periods of anoxia and be favourable to plant growth in more hydric ecosystems (see also Knapp *et al*., 2008). The impacts of extreme precipitation events are often exacerbated by their association, in most climatic regions, with extreme wind storms/cyclones.

Ice storms are a form of extreme precipitation that occurs when liquid precipitation (often in a supercooled state) freezes shortly after contact with the terrestrial surface. The growing layer of ice can add substantial weight to vegetation and therefore result in the loss of branches, limbs, or uproot entire trees (Bragg *et al*., 2003; McCarthy *et al*., 2006; Sun *et al*., 2012).

Extreme wind storms and tropical cyclones are often associated with extreme precipitation events, but have the additional potential to cause, depending upon their intensity severe damage and direct concurrent impacts on the carbon cycle (Fig.3d) *via* defoliation, damage to branches, and windthrow or flooding by (e.g. saltwater) storm surges related tree mortality (Conner & Inabinette, 2003; MCPFE, 2007; Chambers *et al*., 2007; Imbert & Portecop, 2008; Zeng *et al*., 2009; Negrón-Juárez *et al*., 2010a) and lodging in agroecosystems (when crop stems are broken and crops are flattened). In addition, in forests, windthrow can cause long-term indirect lagged impacts on the carbon balance via tree mortality and dry dead wood accumulation that may facilitate lagged insect outbreaks or massive fires (Fig.3d; see also below). Individual extreme storms and cyclones can severely impact the regional carbon balance (e.g. Lindroth *et al*. (2009) for Europe or Chambers *et al*. (2007) for the U.S.). For example, in October 2005, Hurricane Wilma made landfall over the Yucatán peninsula with particularly intense winds. Immediate reductions in leaf area and productivity were observed, while in the year following the hurricane, increased carbon emissions from soils were observed that were attributable to the addition of nitrogen-rich organic matter (Vargas, 2012). Depending on the spatial and tempora l scale considered, the frequency and intensity of the storm/cyclone, the characteristics of the impact and the recovery processes involved, the overall carbon balance can vary between a source and a sink (Fig.2; see e.g. Fisk *et al*., 2013).

Soil erosion can be caused by the extreme precipitation events and extreme wind storms (or a combination of both) and is codetermined by topography, soil characteristics, vegetation cover and human activities (e.g. Lal *et al*., 2013) with significant on- and off-site impacts. Extreme weather events can result in direct, rapid and substantial local soil carbon losses (Hilton *et al*., 2008; Jung *et al*., 2012), and subsequent transport/redistribution and deposition (Goldsmith *et al*., 2008). Soils are especially susceptible to erosion if vegetation cover is low, for example crop ecosystems at fallow stages or grasslands after drought periods. Soil carbon loss due to erosion can therefore be a direct concurrent as well as an indirect lagged climate extreme impact (see Fig.4). In addition, soil erosion leads to losses of soil nutrient and water retention capacity, and to a generally lower productivity on eroded soils (Lal & Pimentel, 2008), inducing further (indirect) lagged impacts on the ecosystems carbon cycle. Eroded soil and mobilized soil organic matter are often redeposited within the same ecosystem at short-timescales, but soil organic carbon can also be laterally exported from a particular ecosystem (VandenBygaart *et al*., 2012; Berhe & Kleber, 2013). The deposition and subsequent residence time of carbon removed with eroded soil determines the contribution of soil organic carbon erosion to CO_2_ fluxes (van Oost *et al*., 2007; Lal & Pimentel, 2008). Soil erosion processes can also increase the terrestrial carbon sink if eroded carbon is not transformed to CO_2_, but trapped in deposits with longer residence times than the original soil (van Oost *et al*., 2007; Hilton *et al*., 2008). Hence, erosion and subsequent sedimentation affects the overall land carbon budget, but the net effect of erosion on the carbon cycle remains controversial (Lal, 2009) and improved, scientifically rigorous terminology may be needed to describe landscape soil carbon turnover (Berhe & Kleber, 2013).

## Indirect impacts

While extreme droughts, heat waves, frosts, precipitation and wind storms are climate extremes, soil erosion can be a direct concurrent impact of extreme precipitation and/or wind storms and, additionally, may be amplified by indirect lagged climate extreme impacts (cf. Fig.4); fires and pest and pathogen outbreaks are impacts facilitated by climate extremes (cf. Fig.4), but initiated by another trigger (not necessarily an extreme event *per se*) (cf. Fig.1b and d).

Fire-related losses of biomass or soil organic matter generally occur as an indirect, and often lagged, impact of climate extremes (cf. Figs3b, d and 4) and are caused by the interaction between biotic (e.g. fuel load) and abiotic factors (e.g. dry weather, wind velocity, fuel continuity, slope of terrain and landscape fragmentation) and human ignition (Moriondo *et al*., 2006; Bowman *et al*., 2009; Aldersley *et al*., 2011; Pausas & Paula, 2012). Fire frequency and intensity are highly sensitive to climate extremes because fire behaviour responds immediately to fuel moisture, which is affected by the combination of precipitation, relative humidity, air temperature and wind speed (Moriondo *et al*., 2006). Fires release carbon stored in biomass and organic soils to the atmosphere in form of CO_2,_ CO, CH_4_ and other climate relevant trace gases and aerosols, but can also serve to prevent land–atmosphere CO_2_ fluxes when burned organic matter (i.e. charcoal) is formed during the combustion process. Charcoal is typically more resistant to decomposition and is thought to contribute to long-term carbon sequestration in soils (Preston & Schmidt, 2006; Schmidt *et al*., 2011), although recent advances point to a much faster decomposition rate which depends on thermal conditions during formation and soil conditions afterwards, than previously thought (Major *et al*., 2010; Singh *et al*., 2012; Kasin & Ohlson, 2013).

Extreme fire events release large quantities of carbon to the atmosphere (Page *et al*., 2002) and may have long-lasting consequences on vegetation composition (Bond *et al*., 2005), soil structure, hydrophobicity and nutrient availability (Certini, 2005) with presumable multiple indirect and lagged impacts on the terrestrial carbon cycle (cf. Figs3b and 4). Carbon stored in litter, and organic soils such as peat, is burned during high-intensity but slow-spreading fires, and can be irreversibly destroyed, particularly during peat fires where carbon accumulated over very long timescales is immediately released, but can be additionally accelerated by another trigger (Page *et al*., 2002; Turetsky *et al*., 2011a). Note, however, that not all climate-induced fires are carbon cycle extremes, but are within the range of the particular disturbance regime. For instance, frequent and low-intensity savannah fires (Archibald *et al*., 2012) may release over a year as much CO_2_ as would have been decomposed otherwise by microbes (Li *et al*., 2013).

The occurrence, frequency and magnitude of insect and pathogen outbreaks are often related to natural cycles in population size, driven by predator-prey type dynamics (Jepsen *et al*., 2009; Kausrud *et al*., 2012). But there is consensus – despite many uncertainties – that climate conditions influence strength and timing of insect/pathogen outbreaks *via* changes in dispersal, reproduction, development of host plants, and mortality and distributional range changes of insect herbivores (Netherer & Schopf, 2010; Cornelissen, 2011). Different types of climate extremes may therefore catalyse insect and pathogen outbreaks leading as we hypothesize towards indirect lagged impacts on the carbon cycle (see Figs3 and 4). Warm temperatures appear to favour radical increases in insect populations as a result of reduced mortality during the cold season, accelerated insect development rates and earlier flight periods (Virtanen *et al*., 1998; Stahl *et al*., 2006; Robinet & Roques, 2010; Johnson *et al*., 2010). We regard these patterns as an indirect lagged impact of fewer cold temperature extremes (cf. Figs3a and 4). Mechanisms, associated with indirect lagged impacts of extreme heat and drought (cf. Figs3b and 4), were observed during the European 2003 heat wave. Soil water deficits appeared to lower tree resistance to pest attacks, that is a positive drought – disease association, and defoliators additionally benefitted from increased nitrogen in plant tissues linked to moderate or intermittent drought stress (Desprez-Loustau *et al*., 2006; Rouault *et al*., 2006). Multiple examples of how primary productivity and carbon stocks are reduced by insects and pathogens, and changes in carbon sink strength, are given in Hicke *et al*. (2012).

## Impacts of extreme events on different ecosystem types

Ecosystems react differently to climate extremes: therefore, we deduce that a climate extreme of a given magnitude will not have the same impact in a forest, grassland, peatland or cropland. With both large aboveground carbon stocks (standing biomass) and carbon uptake being affected by climate extremes, we expect the largest net effects on the terrestrial carbon balance in forests compared to other ecosystems. Forest carbon stocks may be lost or reduced as CO_2_ rapidly by fire (as an indirect concurrent or lagged effect due to drought and heat extremes; Fig.4), or more slowly during the decomposition of dead wood after extreme wind and ice storms or forest dieback after an extreme drought, which lead to lagged carbon emissions for a presumable long period after the climate extreme has occurred.

There are notable differences in how individual tree species respond to intra-annual climatic extremes including the timing of maximum sensitivity (Babst *et al*., 2012), and the complexity of forest ecosystem dynamics makes prediction of the impacts of extreme events on carbon cycling challenging (Rammig *et al*., 2014). At the same time, we hypothesize the complexity of forest ecosystems contributes to their resilience to climate extreme related impacts as, for example heterogeneous forests are known to be less susceptible to windthrow (Lindroth *et al*., 2009), insect outbreaks (Drever *et al*., 2006) and mass movements (Bebi *et al*., 2009) (see Appendix S1, section A. for biome-specific extremes and related impacts). Forests generally have better access to deeper ground water than grasslands and are reported to be likely less strongly affected by drought and heat waves (Teuling *et al*., 2010). However, once their mortality thresholds are passed, we suppose forests to be less resilient to extreme events than grasslands, which have evolved to recover rapidly from disturbances. Natural grasslands prevail in regions where climatic constraints limit the occurrence of woody life forms (Suttie *et al*., 2005). Grasslands are typically characterized by comparatively higher turnover rates compared to woody vegetation, and we therefore assume grasslands to be more resilient to climate extremes than forests (see Appendix S1, section B, for more details). In this context, amongst the climate extremes, drought is expected to have the largest effect on the carbon cycle of grasslands (Zavalloni *et al*., 2008; Gilgen & Buchmann, 2009; van der Molen *et al*., 2011), while other extremes (e.g. wind storms) play a smaller if not negligible role (Reichstein *et al*., 2013). However, degradation feedbacks, as triggered by, for example grazing pressure (Albertson *et al*., 1957), erosion (Breshears *et al*., 2003) or fire combined with extreme precipitation events, may amplify effects of extreme drought and lead to substantial soil carbon losses. In comparison with forests, when normalizing for the per cent of bare soil, potential postfire erosion tends to be lower in grassland (Johansen *et al*., 2001).

Peatlands have characteristics in common with both forests and grasslands, namely large organic carbon stocks and a clear dominance of belowground carbon stocks, respectively. The large carbon stocks stored in peatlands are mainly protected by decomposition-limiting low temperatures and/or high water levels (Freeman *et al*., 2001). Peatland carbon stocks are highly susceptible to immediate oxidation by fire (van der Werf *et al*., 2008, 2010; Turetsky *et al*., 2011a,b) and drought- or drainage-induced processes of microbial decomposition of organic carbon (Jungkunst & Fiedler, 2007; Couwenberg *et al*., 2010; Frolking *et al*., 2011). Therefore, we hypothesize peatlands to be highly susceptibility to drought extremes and fire events caused by climate extremes (see Appendix S1, section C for more details).

Croplands are distinct from forests, grasslands and peatlands, in that most crops are planted and harvested on an annual basis. The response of croplands is strongly coupled to the timing of the climate extreme, that is the sensitivity of the growth stage of the impacted crop (e.g. van der Velde *et al*., 2012) and the management actions taken (e.g. Porter & Semenov, 2005; Ramankutty *et al*., 2008; van der Velde *et al*., 2010; Lobell *et al*., 2012). In croplands, many climate extreme impacts can (theoretically) be mitigated through management, either within the same year (e.g. irrigation, replanting of a failed crop), or through longer term adaptation (e.g. changed rotations, drought- and/or heat-resistant cultivars). Lagged impacts of more than one year are of minor importance in croplands compared with the other ecosystem types.

A quantitative and systematic assessment of the impacts from different types of extreme events is currently limited by the number of observed case studies, a general lack of systematic data, and a lack of common metrics across experimental and impact studies (see Introduction). It is therefore currently only possible to provide a detailed literature survey about how drought, wind storms, temperature and precipitation extremes, may possibly act on carbon cycle processes in forests, grasslands, peatlands and croplands (see Appendix S1).

## Future climate extremes and their impact on the carbon cycle

There are inherently few data available to make robust assessments regarding changes in the frequency or intensity of carbon cycle extremes. First of all, climate extremes are hard to predict, as many predictions of climate extremes are either not sufficiently well resolved (e.g. heavy precipitation) or associated with high uncertainties (e.g. drought) in current climate models (Seneviratne *et al*., 2012). Even in leading sectorial (e.g. agriculture) models, the effects of high temperatures, increased climate variability and several other growth-limiting factors such as soil nutrients, pests and weeds are not yet fully understood, and thus not implemented (Soussana *et al*., 2010). Hence, it is very difficult to anticipate future impacts of climate extremes on the global carbon cycle. Thus, we here only hypothesize the most important current and future risks of the terrestrial carbon cycle in the face of climate extremes given the available literature.

In those parts of the boreal zone where litter and soil moisture will likely decrease, for example *via* rising temperatures and decreasing precipitation (Seneviratne *et al*., 2012) and earlier snowmelt (Grippa *et al*., 2005), we hypothesize an increased risk that extreme dryness and tree mortality will increase the susceptibility to triggers such as lightning and human ignition, causing fires as an indirect concurrent or lagged effect (c.f. Figs1d and 4; Michaelian *et al*., 2011).

On the other hand, according to current climate projections, large areas in the boreal zone will likely become wetter (IPCC, 2013). More extreme snow fall has the potential to lead to stronger insulation of the soil in the winter. The higher soil temperatures may favour the thawing of permafrost (Zhang *et al*., 2001; Gouttevin *et al*., 2012), but also increase mineralization and growing season productivity (Monson *et al*., 2006). Assessment of the magnitude and timing of these two opposing effects will require further research. As host–pathogen interactions are strongly influenced by weather and climate, we further hypothesize that decreased frost occurrence and fewer cold extremes will facilitate pest and pathogen outbreaks (e.g. Virtanen *et al*., 1998; Hicke *et al*., 2012; Sambaraju *et al*., 2012; Price *et al*., 2013) with supposed important indirect and lagged impacts on the carbon cycle.

Temperate regions, being situated between cold boreal and warm, summer-dry Mediterranean regions are susceptible to temperature and precipitation extremes, droughts and storms, and impacts facilitated by them. Storms are considered to be the most important natural disturbance agent in temperate European forests, and even a small increase in storm frequency could potentially lead to a long-term reduction of the carbon stock (Fuhrer *et al*., 2006; Lindroth *et al*., 2009). Yet, current predictions of changes in storm intensity and frequency are not very robust (IPCC, 2013), such that no speculation on future impacts of storms on ecosystem is possible.

In contrast, we conjecture that in dry temperate regions, there will be a sizeable negative effect on the carbon cycle through drought extremes, because towards the drier border of temperate regions, there is consensus among climate models that, for example, the number of consecutive dry days will increase (Seneviratne *et al*., 2012). Droughts, often occurring in concert with heat waves, can extend spatially across subcontinental domains and have a pronounced effect on forests, grasslands and croplands (Reichstein *et al*., 2007; Schwalm *et al*., 2010). Yet, the potentially mitigating effect of increased plant water use efficiency through increased CO_2_ concentrations needs to be scrutinized in future research (e.g. Morgan *et al*., 2011; Zscheischler *et al*., 2014c).

Mediterranean and subtropical ecosystems are already shaped by strong seasonality of water availability. Changes in precipitation patterns with longer dry spells and more intense precipitation events are very likely (Seneviratne *et al*., 2012). We suggest that in forests these changing patterns will contribute to higher tree mortality rates, increased fire activity in forests, and thus more sparse vegetation, and therefore as an indirect lagged effect (cf. Figs1d and 4) enhanced soil erosion, with expected negative consequences for ecosystem productivity (e.g. Allen *et al*., 2010; Williams *et al*., 2012). We further hypothesize that such positive feedback loops within the ecosystem triggered and enforced by alternating dry spells and subsequent heavy precipitation are even more likely and rapidly to occur in grasslands and cropland (e.g. with lower thresholds) because the nonwoody vegetation with shorter turnover is likely to respond faster.

In the tropics, susceptibility of the carbon cycle to climate extremes will strongly depend on the interaction with human drivers. For example, fire risk is low in undisturbed Amazonian rainforests, and almost all fires are a consequence of land-use-related burning activities (Aragão & Shimabukuro, 2010). Once burnt, forests are more susceptible to repeated burning, creating a positive feedback, which has the potential to transform large parts of rainforests into degraded forests or even savannah (Barlow & Peres, 2008; Brando *et al*., 2012, 2014; Morton *et al*., 2013). Changes in precipitation patterns with longer dry spells might additionally increase fire risk with decreasing canopy closure. While tropical forests and cropping systems are susceptible to long-term droughts, heavy precipitation and wind storms, future projections of these climatic extremes are particularly uncertain. The effect of high temperatures on photosynthesis is the second crucial mechanism that can directly impact tropical forests, where the most intensive CO_2_-emission scenarios yield temperatures sufficient to damage photosynthesis and growth (Doughty & Goulden, 2008). But the long-term acclimation and adaptation potential of tropical forest ecosystems (e.g. shift to heat-tolerant species) is not well known (Corlett, 2011; Smith & Dukes, 2013). We expect also the susceptibility of tropical peatlands to climate extremes to be strongly dependent on the interaction with human drivers, as peatland carbon stocks are highly susceptible to fires and drought- or drainage-induced microbial decomposition processes of their organic carbon stocks (see section above). Thus, we hypothesize that climate extremes will affect the tropical rainforest and peatland carbon cycle substantially, but the magnitude will strongly depend on the local human influence on these carbon stocks.

## Outlook: On improving detection and prediction of global carbon cycle extremes

**Figure 5:**
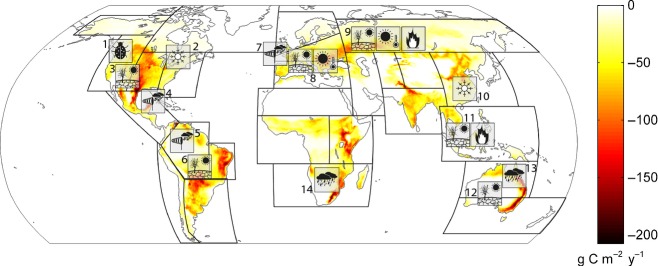
Global distribution of extreme events in the terrestrial carbon cycle, and approximate geographical locations of published climate extremes with impacts on the carbon cycle. Extreme events in the carbon cycle are defined as contiguous regions of extreme anomalies of GPP during the period 1982–2011 (modified after Zscheischler *et al*., 2014b). Colour scale indicates the average reduction in gross carbon uptake compared to a normal year due to negative extremes in GPP. Units are gram carbon per square metre per year. The map highlights the IPCC regions with the following references to the published climate extremes. References: 1 *pest outbreaks Canada/North America* (Soja *et al*., 2007; Kurz *et al*., 2008b), 2 *ice storm North America* (Irland, 2000), 3 *drought US* (Breshears *et al*., 2005; Schwalm *et al*., 2012), 4 *heavy storm Southern US* (Chambers *et al*., 2007; Zeng *et al*., 2009; Negrón-Juárez *et al*., 2010a), 5 *heavy storm Amazon* (Negrón-Juárez *et al*., 2010b), 6 *drought Amazon* (Tian *et al*., 1998; Phillips *et al*., 2009; Lewis *et al*., 2011), 7 *heavy storm Europe* (Fuhrer *et al*., 2006; Lindroth *et al*., 2009), 8 *drought and heat extreme Europe* (Ciais *et al*., 2005; Reichstein *et al*., 2007), 9 *extreme drought, heat and fire in Russia* (Barriopedro *et al*., 2011; Konovalov *et al*., 2011; Coumou & Rahmstorf, 2012; Bastos *et al*., 2013a), 10 *ice storm China* (Stone, 2008; Sun *et al*., 2012)), 11 *fire, drought SE Asia* (Page *et al*., 2002; Schimel & Baker, 2002), 12 *drought Australia* (Haverd *et al*., 2013), 13 *heavy precipitation Australia* (Bastos *et al*., 2013b; Haverd *et al*., 2013), 14 *heavy precipitation Southern Africa* (Bastos *et al*., 2013b).

From a mechanistic and process perspective, it is clear that climate extremes can have a profound impact on the carbon cycle, and case studies have reported such impacts (Fig.5). However, great challenges remain for both a rigorous global quantification of carbon cycle extremes and estimation of the future impacts on terrestrial-atmosphere CO_2_ fluxes, and hence carbon cycle climate feedbacks.

Remote sensing of the biosphere from space with a short return interval to identical locations and nearly global coverage offers promising perspectives to detect extreme anomalies in the biosphere in a consistent way (but see below). Land surface states can be estimated by analysing the interaction of electromagnetic radiation (from visible to microwave) with the vegetation or upper centimetres of the soil *via* relatively well-evaluated radiation transfer models and their inversion. Thus, vegetation states (e.g. leaf area index, biomass) and radiative properties (e.g. fractions of absorbed radiation) can be monitored, albeit they require improvements to correct retrieved signals affected by noise and biases related to atmospheric conditions. Direct methods exist for use on the ground (Pan *et al*., 2011; Baldocchi *et al*., 2012; Babst *et al*., 2014) and can be combined with remote sensing and modelling approaches to infer carbon cycling at the global scale (Jung *et al*., 2011).

Zscheischler *et al*. (2013) have taken a first approach to detect extreme changes in fAPAR (fraction of absorbed photosynthetically active radiation) and GPP (Zscheischler *et al*., 2014a) associated with climate anomalies that occurred during the last three decades and their association with climate anomalies. They presented four major findings: (1) the total effect of negative carbon cycle GPP extremes is of a similar magnitude as the mean terrestrial carbon sink, (2) the spatial distribution of extremes is highly uneven with ‘hotspot’ regions in many semiarid monsoon-affected regions, (3) the distribution of extreme carbon impacts follows a power law and (4) the detected carbon cycle extremes are statistically mostly strongly associated with droughts. The background map in Fig.5 shows the spatial distribution of carbon cycle extremes detected in the Zscheischler *et al*. studies. Many regions, where case studies have reported carbon cycle extremes, are also detected by the global remote sensing-based approach, but not all. In particular, Amazonian extreme anomalies in the carbon cycle suggested by Phillips *et al*. (2009) or Negrón-Juárez *et al*. (2010b) are not evident in the remote sensing-supported analysis of Zscheischler *et al*. (2013) and are only seen in one model in the analysis of negative extremes in four different data-driven and modelled GPP estimates (Zscheischler *et al*., 2014a). One reason for this might be the lack of sensitivity of fAPAR in dense evergreen vegetation (data-driven estimates of GPP often rely strongly on fAPAR). Evergreen vegetation often changes its physiology without strong alterations in the leaf or canopy reflective properties. This effect has also been observed outside tropical regions, for instance, during the extreme heat and drought in Europe 2003 (Reichstein *et al*., 2007). Currently, more direct observations of photosynthetic processes *via* fluorescence offer the potential to overcome this problem (Frankenberg *et al*., 2011; Guanter *et al*., 2014), as well as combined observations of greenness indices and land surface temperature (Mildrexler *et al*., 2009). However, one striking feature of Fig.5 is the lack of presumably reported extreme impacts on the carbon cycle in some hotspot areas seen by the satellite data analysis. These include North East Brazil, the Indian subcontinent, East Asia, and particularly sub-Saharan Africa. To our understanding, without observations and experiments in those tropical hotspot areas, it will be hard to fully understand climate–carbon cycle feedbacks and the role of carbon cycle extremes therein at a global scale.

According to our understanding, not all climate extremes cause extreme impacts in ecosystems, but they can have in-/direct and/or immediate/lagged effects. Lagged effects can either slow down the carbon cycle, when reduced vegetation productivity and/or wide-spread mortality after an extreme drought are not compensated by regeneration, but they can also accelerate the carbon cycle, when, for example productive tree and shrub seedlings cause rapid regrowth after windthrow or fire. Likewise, not all terrestrial carbon cycle extremes are propagated immediately into the atmosphere. For example, an extreme mortality event increases coarse woody debris, which is then slowly decomposed during the following years. Terrestrial carbon cycle extremes leading to structural changes without immediate fluxes to the atmosphere are currently globally undetectable due to lack of observation capabilities. LiDAR or Radar satellite missions with sufficient spatial and temporal resolution should be encouraged to increase such capabilities in the future. Detection systems need to resolve processes that cause immediate or lagged effects at different spatial and temporal scales, as the resilience of the respective ecosystem differ by ecosystem type.

This review also showed the lack of quantitative and consistent experimental data on the impact of climate extremes on the terrestrial carbon cycle, such that our conclusions are largely based on expert knowledge, scattered case studies and logical reasoning. Future experimental and observational designs should have a clear definition of the extreme conditions at the onset (e.g. by return interval), a consistent classification of resulting (extreme) impacts and should consider testing hypotheses around the conceptual framework presented in Fig.1. In particular, indirect effects (Fig.1b and d) need to receive increased attention in our opinion, given the complexity of the mechanisms involved and the paucity of current studies.

Future experiments should not only strive towards increasing comparability of treatments across case studies, as suggested above; they should also account for increasing severity of future climate extremes and test more explicitly for threshold effects and mortality and recovery responses after extreme events, including those related to changing shifts of ecosystem states (Smith, 2011, Beier *et al*., 2012; Bahn *et al*., 2014). Gradient studies that contain at least one very extreme (and possibly unrealistic) treatment would be particularly useful for this (Kreyling *et al*., 2014). Future experiments should address lagged and legacy effects more consistently, as well as ecosystem responses to multiple subsequent climate extremes, with the aim of elaborating mechanisms, as, for example related to stress physiology, mortality and community assembly, as well as plant–soil interactions and soil processes at large (Backhaus *et al*., 2014; Kopittke *et al*., 2014; Vicca *et al*., 2014). Only through holistic approaches will we be able to fully understand the impacts of climate extremes on ecosystem carbon cycling; information needed to obtain realistic predictions of future carbon cycling and climate feedbacks. For more details and best-practice guidance in climate change experiments that aim to improve our understanding of the impacts of climate extremes, we refer to Beier *et al*., 2012; Vicca *et al*., 2012, 2014; Kreyling *et al*., 2014.

For ecosystems dominated by long-lived species such as forests, a better integration of experimental and modelling studies is needed, with experiments targeting critical hypotheses underlying model assumptions or specific mechanisms (e.g. processes linked to ecosystem transitions). State-of-the-art coupled climate–carbon cycle models (CMIP5) indicate a stronger negative effect of carbon cycle extremes than the above-mentioned observation driven estimates (Reichstein *et al*., 2013), and an increasing absolute effect in the future. However, a reliable projection of the future impact of climate extremes on the terrestrial carbon cycle must rely on improved earth-system modelling, as well as improved description of the biospheric responses. Higher spatial (both horizontal and vertical) resolution and better representation of convective processes and clouds are prerequisites for the simulation of climate extremes, and particularly hydrometeorological extremes. On the biosphere modelling side, all processes leading to direct/indirect, as well as concurrent/lagged impacts (Fig.4), need to receive attention. In particular, vegetation mortality in response to climate extremes (e.g. drought) and its mechanisms are increasingly well documented. Effort needs to be taken now to include this knowledge into global biosphere models. Including pest and pathogens, their reaction to climate extremes such as cold extremes and their effect on the carbon cycle within an integrated modelling system at global scale is likely still too ambitious and needs landscape-modelling approaches, where lateral interactions are considered. Promising local- to regional-scale approaches do exist here and need to be further developed (Seidl *et al*., 2011). Representation of these impacts into carbon cycle models will likely increase projected effects of climate extremes on the carbon cycle. On the other hand, we have to note that fundamental adaptive processes, such as acclimation, plasticity, migration, selection and evolution have the potential to mitigate effects of climate extremes. Modelling approaches accounting for these adaptations urgently need to be underpinned with more observational data and further developed (Scheiter *et al*., 2013).

This study underlines the demand for better structured impacts studies of climate extremes on terrestrial ecosystems and the carbon cycle which follow a standardized protocol and definitions and allow for intercomparison studies. It has also shown the varying depth of analysis for different types of climate extremes, as well as identifying critically understudied regions. The findings underline the importance of biospheric processes in modulating impacts of climate extremes to assess the feedback to the global carbon cycle. In other words, biospheric processes are likely to determine the reaction of the global carbon cycle to climate extremes under global change.

## Supporting Information

**Appendix S1.** Provides a detailed literature survey about how climate extremes act on forests, grasslands, peatlands and croplands.
