# Supplementary material for: Effects of climate extremes on the terrestrial carbon cycle: concepts, processes and potential future impacts
Source: Glob Chang Biol. 2015 May 12;21(8):2861–80. doi: 10.1111/gcb.12916 (PMC4676934; doi:10.1111/gcb.12916)
Supplement: Supplementary file 1 [file gcb0021-2861-sd1.docx]

**Supporting Information** Appendix S1

Supporting information S1 provides a literature survey about how climate extremes may possibly act on forests (A), grasslands (B), peatlands (C) and croplands (D).

**A. Forests**

Forests cover about 30% of the global land area and play an important role in the global carbon cycle (Canadell & Raupach, 2008; FAO, 2010; Pan *et al.,* 2011). In addition to their large carbon pools and fluxes, characteristics of forests that likely make them susceptible to climate extremes, in terms of the terrestrial carbon cycle, include:

- the long lifespan of individual organisms that store carbon in living tissues, structural and hydraulic infrastructures
- location of meristems where (re)growth can happen after an extreme event,
- a high sensitivity to diverse climatic extremes at multiple spatiotemporal scales
- lagged physiological and cascading ecological processes leading to long-term changes in growth and/or mortality
- high vulnerability of carbon stocks and long recovery time to re-gain previous stocks following extreme event impacts
- low migration rates in response to environmental changes compared to short-living species

We hypothesize drought to be the primary control on both inter-annual variability of forest productivity and long-term tree survival. In trees, vulnerability to drought-induced hydraulic failure (Choat *et al.,* 2012, but see Klein *et al.,* 2014) may have both concurrent and lagged (often carbohydrate and mortality related) consequences for the carbon cycle. Besides hydraulic failure, tree mortality following drought and heat waves has been suggested to be caused by carbon starvation and / or cellular metabolism limitation (Adams *et al.,* 2009; Sala *et al.,* 2010; McDowell *et al.,* 2011, 2013). Repeated stress from one or multiple extreme climate events in combination with non-climatic disturbances can lead to a long-term recovery-response of a forest or to a downward spiral into decline and mortality thereby enhancing negative impacts of drought on the carbon sequestration potential (Rouault *et al.,* 2006; Sánchez-Salguero *et al.,* 2012).

Similar to temperate regions, drought is a significant driver of the physiology and carbon cycling of Mediterranean (Granier *et al.,* 2007; Schwalm *et al.,* 2012) and tropical forests (Tian *et al.,* 1998; Phillips *et al.,* 2009; Clark *et al.,* 2010). Tropical wet forest tree growth was found to be highly sensitive to the current range of dry season conditions and to moderate (1–2°C) variations in mean annual night-time temperature (Clark *et al.,* 2010). The severe droughts in the Amazon during 2005 and 2010 caused a significant large-scale increase in tree mortality with an estimated committed biomass carbon loss of up to 1.6 Pg C and 2.2 Pg C (Phillips *et al.,* 2009; Lewis *et al.,* 2011). However, a recent analysis (Cox *et al.,* 2013) suggests stronger resilience to warming than previously (Cox *et al.,* 2004) reported and radiocarbon evidence for living trees older than 1000 years demonstrates that trees have survived though several mega-El-Niño type droughts during the past millennium (Chambers *et al.,* 1998). Thus to our understanding it remains unclear how vulnerable and how close the Amazon or other tropical forests, which may be even more sensitive to drought (Meir & Woodward, 2010), are to climate driven biome shifts.

The 2003 European heat wave strongly affected the hydraulic balance in many tree species with symptoms ranging from partial crown necrosis to death (Martinez-Meier *et al.,* 2008, Eilmann *et al.,* 2009, 2011), whereas a positive growth response was observed at higher elevations across the Alps (Jolly *et al.,* 2005). Also, in boreal ecosystems, a positive temperature extreme during the growing season tends to result in enhanced productivity (Esper *et al.,* 2002; Babst *et al.,* 2013), but a positive temperature extreme occurring during the winter will most likely tend to increase respiration, and therefore result in a loss of carbon from the ecosystem (Piao *et al.,* 2008).

Heavy snow fall may result in crown, stem breakage, and even fully uproot trees. Nykänen *et al.* (1997) estimated an average annual timber loss of 4x106 m³ across Europe due to snow damage. Spring and autumn snowfall (when deciduous trees still hold foliage) and snow temperatures around 0°C (high water content and enhanced abilities to accumulate) increase the likelihood of damage (Nykänen *et al.,* 1997) as do higher wind speeds (Valinger & Fridman, 1999).

Storms are considered to be the most important natural disturbance agent in temperate European forests and even a small increase in storm frequency could potentially lead to a long-term reduction of the carbon stock (Fuhrer *et al.,* 2006). The large storm “Lothar” in central Europe killed the equivalent of 16 Tg C tree biomass (Lindroth *et al.,* 2009), and hurricanes Katrina and Rita destroyed 43.9 ± 8.4 Tg C and 37.9 ± 6.4 Tg C of living biomass, respectively (Negrón-Juárez *et al.,* 2010). Katrina alone caused a total biomass loss estimated around 50–140% of the net annual U.S. carbon sink of forest trees (Chambers *et al.,* 2007).

Forest fires released around half of the average annual 2.0 Pg C globally emitted by fires between 1997 and 2009 (van der Werf *et al.,* 2010). Fire regimes are regarded as highly non-linear, where under extreme climate conditions the burnt area can increase by an order of magnitude (Sukhinin *et al.,* 2004; Vivchar, 2011). Lagged effects due to precipitation deficits overlaid with spring droughts caused extreme fire events in Siberian permafrost regions (Forkel *et al.,* 2012).

Outbreaks of forest insects damage around 35x10^6^ ha of forest annually primary in the temperate and boreal zones (FAO, 2010). Climate extremes may impact the outbreak strength, timing and frequency as well as the host plant resistance. The European 2003 heat wave showed the importance of soil water status in tree resistance against pest attacks (c.f. Desprez-Lousteau *et al.,* 2006; Rouault *et al.,* 2006). In north-western North America favourable climate conditions, such as e.g. reduced minimum winter temperature (i.e. less cold extremes, cf. Fig. 3a, Fig.4), have resulted in the most extensive and severe bark beetle damages ever reported (Kurz *et al.,* 2008). Vast expanses of forest have become a net carbon source and this is predicted to persist at least until 2020.

B. Grasslands

Grasslands are characterized by high turnover rates and a rapid recovery from disturbance. Amongst the climate extremes, drought has the presumably largest effect on the carbon cycle of grasslands, with substantial implications also for society. For example, during the 2003 summer drought in Central Europe, grassland production ceased completely in certain areas, resulting in an annual decrease of fodder production across Europe between 30% and 60% (EEA, 2005). Typically, drought effects are higher for above-ground productivity (e.g. Kahmen *et al.,* 2005; De Boeck *et al.,* 2011; Fay *et al.,* 2011) than for below-ground productivity, resulting in changes in carbon allocation belowground and an increased root-to-shoot ratio (e.g., Dukes *et al.,* 2005; Gilgen & Buchmann, 2009; Burri *et al.,* 2014). As for forests, soil respiration of grasslands decreases when soil moisture drops below critical thresholds (Knapp *et al.,* 2002; Bahn *et al.,* 2008; Joos *et al.,* 2010; Burri *et al.,* 2014), with consequences for annual soil respiration (Ma *et al.,* 2007; Bahn *et al.,* 2010). However, rainfall following drought may rapidly and strongly stimulate soil CO_2_ emissions (Borken & Matzner, 2009), and in semi-arid grasslands may cause higher respiratory CO_2_ losses than from the rest of dry season (Xu *et al.,* 2004).

The magnitude and direction of carbon cycle responses to reduced precipitation and drought depend strongly on the climatic context and background soil moisture conditions. While in semiarid and arid climates water availability severely limits ecosystem carbon acquisition (Hunt *et al.,* 2004) and soil respiratory carbon losses, the response in temperate conditions is less clear (Bloor *et al.,* 2010). In humid climates and under very wet conditions, reduced precipitation has even been shown to increase aboveground plant growth and ecosystem carbon uptake (Jaksic *et al.,* 2006; Gilgen & Buchmann, 2009; Peichl *et al.,* 2011).

In addition to the amount, seasonal variability and timing of precipitation and associated drought may strongly alter key carbon cycling processes and plant community composition (Knapp *et al.,* 2002; Harper *et al.,* 2005; De Boeck *et al.,* 2011). In fact, the timing of precipitation may affect carbon cycling even more strongly than changes in rainfall quantity (Chou *et al.,* 2008; Hovenden *et al*., 2013). In dry grasslands, the effects from the combined timing and size of rainfall events are crucial (Thomey *et al.,* 2011) as these factors co-determine critical soil moisture thresholds (Vargas *et al.,* 2012).

Experiments on simplified sown experimental grasslands revealed a high recovery potential of plant growth (Zavalloni *et al.,* 2008; De Boeck *et al.,* 2011). High resilience of key plant ecophysiological processes as well as soil carbon fluxes after prolonged droughts was observed also in managed temperate grasslands (Gilgen & Buchmann, 2009; Joos *et al.,* 2010; Signarbieux & Feller, 2011). Furthermore, changes in vegetation structure and composition in response to drought can contribute to the resilience of grasslands, and may alter above- and belowground productivity and CO_2_ fluxes (including their response functions to climate) beyond the direct physiological response (Kreyling *et al.,* 2008; Talmon *et al.,* 2011; van der Molen *et al.,* 2011), but might also result in increased weed pressure (Gilgen *et al.,* 2010).

Drought may have strong interactive effects with high temperatures, which increase water vapour pressure deficits and the soil water stress, and can amplify detrimental effects of drought on the physiology of organisms, e.g. during heat waves (Chaves & Oliveira, 2004; De Boeck *et al.,* 2011). An anomalously warm year was shown to exert both immediate and lagged effects on the net ecosystem exchange of CO_2_ of a tallgrass prairie (Arnone *et al.,* 2008). This extreme decreased NEE immediately via drought effects on net primary productivity and in the following year by stimulating the respiration of soil heterotrophs. In a future climate, elevated CO_2_ may buffer effects of drought on grassland productivity by increasing water use efficiency (Owensby *et al.,* 1997). The combination of warming, elevated CO_2_ and limiting soil moisture may favour C_4_ over C_3_ grasses and may thereby result in higher productivity in semi-arid grasslands than expected (Morgan *et al.,* 2011).

Heavy rainfall, particularly after prolonged drought periods, can lead to degradation via soil erosion in already degraded grasslands that lack grass cover. While intense rainfall has less pronounced effects on erosion in grasslands and rangelands than in arable lands (van Oost *et al.,* 2007), once degradation feedbacks come into play, more frequent extreme events may contribute to a desertification of semi-arid to arid grassland particularly when (over-)grazing or fire act as an additional pressure. Heavy or prolonged rainfall can also cause water-logging in grasslands, affecting root mortality, forage quality and quantity, and ultimately vegetation composition.

Fires are an important factor of ecosystem dynamics in (sub-)tropical grasslands or savannahs, where the vegetation has adapted and co-evolved re-sprouting traits coinciding to a certain fire frequency (Pausas *et al*., 2004, 2009). Fires in grasslands and savannahs contributed 44% to the total global fire carbon emissions during 2001–2009 due to frequent burning of large areas (van der Werf *et al.,* 2010). Due to vast regeneration of grasslands, fluxes and species composition can return to pre-fire conditions within a few months. Castaldi *et al.* (2010) observed that one month after burning a tropical grassland, CO_2_ emissions were significantly lower in burned plots than in the control plots, but after eight months they no longer differed.

C. Peatlands

Peatlands contain in about 400 Mha between 400 to 600 Pg C (Frolking *et al.,* 2011); the carbon stored in peatlands is protected primarily by the prevailing environmental conditions that limit decomposition: low temperatures and/or high water levels (Freeman *et al.,* 2001), which makes peatlands hotspots in terms of potentially large feedbacks to global change. Peatlands are particularly susceptible to oxidation of the carbon stocks by fire and biological decomposition processes, which may be induced by drought. Fires cause immediate oxidation of large amounts of carbon stored in peat soils (van der Werf *et al.,* 2008; Hooijer *et al.,* 2010), whereas droughts can substantially increase soil carbon efflux following soil aeration (Freeman *et al.,* 2001). Moreover, Sowerby *et al.* (2008) found a persistent stimulation of decomposition rates in a peatland experiment that was exposed to repeated summer droughts, which was confirmed by Couwenberg *et al.* (2010) for South-east Asia and Turetsky *et al.* (2011a) in northern peatlands. This persistent stimulation of soil decomposition was related to incomplete recovery of the soil moisture content upon rewetting, which was most likely due to increased soil hydrophobicity. Hence, legacy effects of drought and also of fire, which can increase soil hydrophobicity (Howell *et al.,* 2006), can substantially increase the impact of a particular extreme event on the large carbon stocks stored in peatlands.

Because of the vast quantities of carbon stored in organic soils (Tarnocai *et al.,* 2009; Page *et al.,* 2011), changes in depth of the water-table from drainage or from drought can expose large areas of carbon to rapid decomposition and decay.

We assume the vulnerability of peatland ecosystems to climate extremes to increase because of human land use change in tropical regions (Hooijer *et al.,* 2010), and because of chronic climate change in high-latitude regions (Turetsky et al 2002; Turetsky *et al.,* 2011b).

D. Croplands

Climate variability and climate extremes strongly affect crop production and thus the long-term carbon balance. Lagged ecosystem impacts of more than one year are of minor importance in croplands; however, lagged impacts do occur due to adaptive changes in the management of arable systems linked to climate extremes and climate change. Indeed, human response to climate extremes, in terms of adaptive management, is one of the greatest uncertainties when attempting to predict the impact of climate extremes on the carbon balance of croplands (Klein Goldewijk & Ramankutty, 2004; Porter & Semenov, 2005; Ramankutty *et al.,* 2008).

Efficient and effective agricultural practice requires that farmers adapt to climate variability. Climate extremes can impact crops via both negative impacts on plant physiological processes and direct physical damage, as well as by affecting the timing and conditions of field operations. The impact of a climate extreme on a certain crop is a function of the timing and type of climate extreme in relation to the sensitivity of the growth stage of the impacted crop (e.g. van der Velde *et al.,* 2012). Even during climate extremes, farmers will pursue strategies to minimize impacts on final crop harvests. Irrigation, for example, can be used during a heat wave to lower ambient field temperature (by ~3-4 °C at an ambient temperature >35 °C), thus minimizing crop heat stress and leading to lower crop losses than would otherwise occur (van der Velde *et al.,* 2010). The use of light-reflecting particle films sprayed over field crops and orchards is another adaptive measure (Glenn & Puterka, 2005). The use of kaolin powder, for instance, can effectively induce radiation loads and excessive heat during extreme events by increasing surface albedo, thus reducing physiological plant damage (Rosati *et al.,* 2006). Adaptive management actions can be immediate, but can also be longer term, such as the use of more heat resistant varieties, or changes in the rotation such as shifting to winter crops (Porter & Semenov, 2005; Lobell *et al.,* 2012; Ramankutty *et al.,* 2008). Adaptive management may lead to co-benefits or trade-offs; Increased irrigation e.g. may lead to increased root biomass growth, higher microbial activity, or increased erosion rates, variously leading to either increased or decreased soil organic carbon levels. Expected crop failure – for instance a crop affected by drought – can also cause a farmer to decide not to harvest the crop, potentially leading to a larger incorporation of biomass into the soil.

Drought stress is one of the most damaging climate extremes affecting crop growth and productivity, especially if it occurs in connection with high temperatures. The 2003 heat wave in parts of Europe reduced crop yields by 20% compared to the mean for 1991-1999 (Fuhrer *et al.,* 2006). Farmers adapt to or mitigate against drought by irrigation. Globally, the area of irrigated land is increasing due to agricultural intensification with consequences for water resources (Nellemann *et al.,* 2009). Introduction of drought resistant crops as well as the use of perennial grain crops with deeper rooting systems (Glover *et al.,* 2010) is another important longer term adaptation strategy.

Long term effects of extreme high temperatures on crop growth and the associated carbon cycle are uncertain due to the complex interaction of different factors (although threshold temperatures for damage of enzymatic reactions and shifts in phenology are well defined; Porter & Semenov; 2005, Lobell *et al.,* 2012). An early effect of heat stress is a change in the photosynthetic pathways following protein damage, reducing the efficiency of photosynthesis. If exposure is long, protein damage and enzyme dysfunction become permanent and lead to irreversible plant damage or death. Effects of extreme high temperatures depend on crop type, variety and genotype, the development stage of the crop at the time of the event, the duration of the event, and its combination with other environmental stressors such as ozone (Fuhrer, 2003). High temperatures shift and/or shorten the phenological development stages, which can also affect yield and biomass production (Larcher, 2003; Porter & Semenov, 2005; Lobell *et al.,* 2011, 2012). Heat stress can also decrease pollination efficiency and grain production by seed abortion, thereby decreasing yields. In addition to day-time temperature effects, crops are also affected by high night-time temperatures, which increases the loss of carbon due to higher respiration rates, and thereby reduces yields in rice and cotton - but uncertainties concerning net carbon storage remain high (Peng *et al.,* 2004; Loka & Oosterhuis, 2010).

Although most of the above-ground biomass in croplands is removed during harvest, biomass production determines the amount of carbon entering the soil with above- and below-ground litter ultimately impacting soil organic carbon levels. However, high temperature can also affect the harvest index, increasing the uncertainty about the net effect of high temperature events (Porter and Semenov 2005). This is important as soil organic carbon represents the only long term carbon storage in croplands (Freibauer *et al.,* 2004; Smith 2004; Smith *et al.,* 2010; Ciais *et al.,* 2010; Smith 2012). Importantly, soil organic carbon is in itself temperature sensitive, which makes the net storage in croplands an uncertain entity (Ciais *et al.,* 2010; Davidson & Janssens, 2006; Schulze *et al.,* 2009).

There are clear indications of the importance of frost damage in affecting changes in the carbon cycle, especially in winter crops (from plant damage to crop failure), but uncertainty remains high and evidence is too sparse to estimate the overall net effect (Bélanger *et al.,* 2002; Gu *et al.,* 2008).

Heavy rain and storm events can destroy above-ground biomass by damage or destruction of the crop, and they can lead to erosion and dislocation of soil, and consequently soil organic carbon loss. Heavy rain can also lead to water logging of soils affecting plant roots, subsequently leading to suboptimal growth and carbon cycling in soils (Fuhrer *et al.,* 2006). Nevertheless, contrasting crop responses to excess precipitation can also occur, for instance in 2007 high summer precipitation rates in France led to low wheat yields but very high maize yields (van der Velde *et al.,* 2012). The excessive rain directly impacted the wheat crop, with excessive soil moisture favouring pest and disease development, increasing lodging, and reducing grain quality (Mars Bulletin, 2007), followed by poor field accessibility hindering wheat harvest. By contrast, maize productivity was not affected and the dry conditions that followed the wet spell at the beginning of September 2007, coupled with high residual soil moisture levels, led to favourable conditions for maize –which in France is not harvested until mid-October (Mars Bulletin, 2007). Adaptive responses to extreme wind experienced during storms can include windbreaks, which can contribute to producer profitability and environmental quality (e.g. Brandle *et al.,* 2004), by protecting cropland fields against extreme wind impacts on crops and soil. During the 20^th^ Century, shorter cereal varieties with a lower risk of lodging, have replaced taller varieties. Longer term adaptive management to high winds and heavy rainfall could involve shorter and sturdier varieties.

Mid-latitude regions (between about 30° and 50°) can experience hail events that may reach extreme intensities with the kinetic energy of hailstones proportional to the 4^th^ power of their diameter (Eccel & Ferrari, 1997). Hail fall may cause severe defoliation in the majority of broad leaf crop species and in corn. Defoliations greater than 90% have been reported for corn and soybeans. Recent observations in the Eastern Alps show that hail intensities have significantly increased by 1.22–1.69% per year from 1975 to 2009 (Eccel *et al.,* 2012). The possibility to predict hail events is low as they may occur under diverse meteorological conditions (Garcıa-Ortega *et al.,* 2011).

Tolerable natural soil erosion rates are generally lower than rates experienced in cropland. Current estimates of soil erosion rates in cropping systems are highly variable and amongst others dependent on slope and tillage practices. Best estimates suggest that soil erosion rates are about two to three magnitudes higher than the rate of soil formation through weathering and dust deposition (Pimentel & Kounang, 1998; Brantley *et al.,* 2007). Single events can have a substantial impact on sediment load over a given time period (e.g. Thothong *et al.,* 2011). Indeed, single exceptional rainfall events can trigger extreme runoff and widespread erosion in areas normally not exposed to a high risk of erosion. In Norway, erosion events (>100 tons ha^-1^) resulted from a combination of extreme rainfall, agricultural management practices, low vegetation cover and a saturated soil overlying a frozen subsoil (Øygarden, 2003). In areas with an increased likelihood of extreme precipitation, erosion and loss of soil and soil organic carbon away from the impacted cropland is a risk. Also in this case, the use of perennial grain crops can be seen as possible adaptive measure, as those crops could potentially reduce the impact of extremes on erosion and soil degradation, NO_3_ leaching, and soil carbon loss (Culman *et al.,* 2013). Their use requires, however, proper assessment of the net balance between the advantage of increased extreme event adaptation and the disadvantage of limited yield levels that can be achieved in the good years (Glover *et al.,* 2010).

Fire is of minor importance with regard to the carbon balance of croplands globally. Agricultural waste burning contributed approx. 3% to the total global fire carbon emissions during 2001–2009 (van der Werf *et al.,* 2010). Reduction in litter input to the soil by burning can reduce soil organic carbon content (Lal, 2007), but the production and incorporation of charcoal in soil may have beneficial impacts (Skjemstad *et al.,* 2002); the net effect remains uncertain.

Climate change – specifically increased temperatures and wetness – is generally thought to spread pests and pathogens beyond their current range, and to introduce alien species into new croplands, as well as increasing their multiplication rates (Gregory *et al.,* 2009). For instance, the survival of *O. poecilus* (rice stink bug) is dependent on the off-season rainfall affected by ENSO (Sutherland & Baharally, 2003). The interactions between crops, pests and pathogens are complex and poorly understood in the context of climate change, but it is clear that crops will be more susceptible to pests and diseases if previously weakened by extreme temperature, drought or other adverse impacts (Gregory *et al.,* 2009). Weed-crop interactions will change, with positive or negative consequences for the crop, while weeds (like crops) can benefit from a positive CO_2_ fertilization effect (Patterson, 1995). In the context of food security, rodent outbreaks associated with an extreme event, cyclone Nargis, impacted agricultural production in Asia (Singleton *et al.,* 2010). The overall impact of pests and pathogens on crops, and the carbon balance of croplands remain uncertain.

**References**

Adams HD, Guardiola-Claramonte M, Barron-Gafford GA *et al.* (2009) Temperature sensitivity of drought-induced tree mortality portends increased regional die-off under global-change-type drought. *Proceedings of the National Academy of Sciences of the United States of America,* **106**, 7063-7066.

Arnone III JA, Verburg PSJ, Johnson DW *et al.* (2008) Prolonged suppression of ecosystem carbon dioxide uptake after an anomalously warm year. *Nature,* **455**, 383-386.

Babst F, Poulter B, Trouet V *et al.* (2013) Site- and species-specific responses of forest growth to climate across the European continent. *Global Ecology and Biogeography,* **22**, 706-717.

Bahn M, Rodeghiero M, Anderson-Dunn M *et al.* (2008) Soil respiration in European grasslands in relation to climate and assimilate supply. *Ecosystems,* **11**, 1352-1367.

Bahn M, Reichstein M, Davidson EA *et al.* (2010) Soil respiration at mean annual temperature predicts annual total across vegetation types and biomes. *Biogeosciences,* **7**, 2147-2157.

Bahn M, Reichstein M, Dukes JS, Smith MD, McDowell NG (2014) Climate-biosphere interactions in a more extreme world. *New Phytologist,* **202**, 356-359.

Bélanger G, Rochette P, Castonguay Y, Bootsma A, Mongrain D, Ryan DAJ (2002) Climate change and winter survival of perennial forage crops in eastern Canada. *Agronomy Journal,* **94**, 1120-1130.

Bloor JMG, Pichon P, Falcimagne R, Leadley P, Soussana J-F (2010) Effects of warming summer drought and CO2 enrichment on aboveground biomass production, flowering phenology, and community structure in an upland glassland ecosystem. *Ecosystems,* **13**, 888-900.

Borken W, Matzner E (2009) Reappraisal of drying and wetting effects on C and N mineralization and fluxes in soils. *Global Change Biology,* **15**, 808-824.

Brandle JR, Hodges L, Zhou XH (2004) Windbreaks in North American agricultural systems. *Agroforestry Systems,* **61-62**, 65-78.

Brantley SL, Goldhaber MB, Ragnarsdottir KV (2007) Crossing disciplines and scales to understand the critical zone. *Elements,* **3**, 307-314.

Burri S, Sturm P, Prechsl UE, Knohl A, Buchmann N (2014) The impact of extreme summer drought on the short-term carbon coupling of photosynthesis to soil CO_2_ efflux in a temperate grassland. *Biogeosciences* **11**, 961–975.

Canadell JG, Raupach MR (2008) Managing forests for climate change mitigation. *Science,* **320**, 1456-1457.

Castaldi S, de Grandcourt A, Rasile A, Skiba U, Valentini R (2010) CO_2_, CH_4_ and N_2_O fluxes from soil of a burned grassland in Central Africa. *Biogeosciences,* **7**, 3459-3471.

Chambers JQ, Higuchi N, Schimel JP (1998) Ancient trees in Amazonia. *Nature,* **391**, 135-136.

Chambers JQ, Fisher JI, Zeng HC, Chapman EL, Baker DB, Hurtt GC (2007) Hurricane Katrina's carbon footprint on U.S. Gulf Coast forests. *Science,* **318**, 1107.

Chaves MM, Oliveira MM (2004) Mechanisms underlying plant resilience to water deficits: prospects for water-saving agriculture. *Journal of Experimental Botany,* **55**, 2365-2384.

Choat B, Jansen S, Brodribb TJ *et al.* (2012) Global convergence in the vulnerability of forests to drought. *Nature,* **491**, 752-755.

Chou WW, Silver WL, Jackson RD, Thompson AW, Allen-Diaz B (2008) The sensitivity of annual grassland carbon cycling to the quantity and timing of rainfall. *Global Change Biology,* **14**, 1382-1394.

Ciais P, Wattenbach M, Vuichard N *et al.* (2010) The European Carbon Balance. Part 2: Croplands. *Global Change Biology,* **16**, 1409-1428.

Clark DB, Clark DA, Oberbauer SF (2010) Annual wood production in a tropical rain forest in NE Costa Rica linked to climatic variation but not to increasing CO_2_. *Global Change Biology,* **16**, 747-759.

Couwenberg J, Dommain R, Joosten H (2010). Greenhouse gas fluxes from tropical peatlands in south-east Asia. *Global Change Biology,* **16**, 1715-1732.

Cox PM, Betts RA, Collins M, Harris PP, Huntingford C, Jones CD (2004) Amazonian forest dieback under climate-carbon cycle projections for the 21st century. *Theoretical and Applied Climatology,* **78**, 137-156.

Cox PM, Pearson D, Booth BB, Friedlingstein P, Huntingford C, Jones CD, Luke CM (2013) Sensitivity of tropical carbon to climate change constrained by carbon dioxide variability. *Nature,* **494**, 341-344.

Culman SW, Snapp SS, Ollenburger M, Basso B, DeHaan LR (2013) Soil and water quality rapidly responds to the perennial grain kernza wheatgrass. *Agronomy Journal,* **105**, 735-744.

Davidson EA, Janssens IA (2006) Temperature sensitivity of soil carbon decomposition and feedbacks to climate change. *Nature,* **440**, 165-173.

De Boeck HJ, Dreesen FE, Janssens IA, Nijs I (2011) Whole-system responses of experimental plant communities to climate extremes imposed in different seasons. *New Phytologist,* **189**, 806-817.

Desprez-Loustau M-L, Marçais B, Nageleisen L-M, Piou D, Vannini A (2006) Interactive effects of drought and pathogens in forest trees. *Annals of Forest Science,* **63**, 597-612.

Dukes JS, Chiariello NR, Cleland EE *et al.* (2005) Responses of grassland production to single and multiple global environmental changes. *Plos Biology,* **3**, 1829-1837.

Eccel E, Ferrari P (1997) La grandine in Trentino: Risultati dell'analisi climatologica per il ventennio 1974-1993. *Quaderni di Esperienze & Ricerche, Istituto Agrario di S. Michele,* **3**.

Eccel E, Cau P, Riemann-Campe K, Biasioli F (2012) Quantitative hail monitoring in an alpine area: 35-year climatology and links with atmospheric variables. *International Journal of Climatology,* **32**, 503-517.

Eilmann B, Zweifel R, Buchmann N, Fonti P, Rigling A (2009) Drought-induced adaptation of the xylem in Scots pine and pubescent oak. *Tree Physiology,* **29**, 1011-1020.

Eilmann B, Zweifel R, Buchmann N, Pannatier EG, Rigling A (2011) Drought alters timing, quantity, and quality of wood formation in Scots pine. *Journal of Experimental Botany,* **62**, 2763-2771.

Esper J, Cook ER, Schweingruber FH (2002) Low-frequency signals in long tree-ring chronologies for reconstructing past temperature variability. *Science,* **295**, 2250-2253.

European Environment Agency (EEA) (2005) *Vulnerability and Adaptation to Climate Change. Technical Report No. 7,* Copenhagen, European Topic Centre for Air and Climate Change (ETC/ACC).

Fay PA, Blair JM, Smith MD, Nippert JB, Carlisle JD, Knapp AK (2011) Relative effects of precipitation variability and warming on tallgrass prairie ecosystem function. *Biogeosciences,* **8**, 3053-3068.

Food and Agriculture Organization of the United Nations (FAO) (2010) Global Forest Resources Assessment 2010. Main Report. FAO Forestry Paper 163. Rome, Food and Agriculture Organization of the United Nations, 340 pp.

Forkel M, Thonicke K, Beer C, Cramer W, Bartalev S, Schmullius C (2012) Extreme fire events are related to previous-year surface moisture conditions in permafrost-underlain larch forests of Siberia. *Environmental Research Letters,* **7**, 044021.

Freeman C, Ostle N, Kang H (2001) An enzymic 'latch' on a global carbon store. *Nature,* **409**, 149-150.

Freibauer A, Rounsevell MDA, Smith P, Verhagen J (2004) Carbon sequestration in the agricultural soils of Europe. *Geoderma,* **122**, 1-23.

Frolking S, Talbot J, Jones MC, Treat CC, Kauffman JB, Tuittila ES, Roulet N (2011) Peatlands in the Earth's 21st century climate system. *Environmental Reviews,* **19**, 371-396.

Fuhrer J (2003) Agroecosystem responses to combinations of elevated CO_2_, ozone, and global climate change. *Agriculture, Ecosystems und Environment,* **97**, 1-20.

Fuhrer J, Beniston M, Fischlin A, Frei C, Goyette S, Jasper K, Pfister C (2006) Climate risks and their impact on agriculture and forests in Switzerland. *Climatic Change,* **79**, 79-102.

García-Ortega E, López L, Sánchez JL (2011) Atmospheric patterns associated with hailstorm days in the Ebro Valley, Spain. *Atmospheric Research,* **100**, 401-427.

Gilgen AK, Buchmann N (2009) Response of temperate grasslands at different altitudes to simulated summer drought differed but scaled with annual precipitation. *Biogeosciences,* **6**, 2525-2539.

Gilgen AK, Signarbieux C, Feller U, Buchmann N (2010) Competitive advantage of *Rumex obtusifolius* L. might increase in intensively managed temperate grasslands under drier climate. *Agriculture, Ecosystems and the Environment,* **135**, 15-23

Glenn DM, Puterka GJ (2005) Particle films: a new technology for agriculture. *Horticultural reviews*, **31**, 1-44.

Glover JD, Reganold JP, Bell LW *et al.* (2010) Increased food and ecosystem security via perennial grains. *Science* **328**, 1638-1639.

Granier A, Reichstein M, Bréda N *et al.* (2007) Evidence for soil water control on carbon and water dynamics in European forests during the extremely dry year: 2003. *Agricultural and Forest Meteorology,* **143**, 123–145.

Gregory PJ, Johnson SN, Newton AC, Ingram JSI (2009) Integrating pests and pathogens into the climate change/food security debate. *Journal of Experimental Botany,* **60**, 2827-2838.

Gu L, Hanson PJ, Mac Post W *et al.* (2008) The 2007 eastern US spring freezes: Increased cold damage in a warming world? *Bioscience,* **58**, 253-262.

Harper CW, Blair JM, Fay PA, Knapp AK, Carlisle JD (2005) Increased rainfall variability and reduced rainfall amount decreases soil CO_2_ flux in a grassland ecosystem. *Global Change Biology,* **11**, 322-334.

Hooijer A, Page S, Canadell JG, Silvius M, Kwadijk J, Wosten H, Jauhiainen J (2010) Current and future CO_2_ emissions from drained peatlands in Southeast Asia. *Biogeosciences,* **7**, 1505-1514.

Hovenden MJ, Newton PCD, Wills KE (2013) Seasonal not annual rainfall determines grassland biomass response to carbon dioxide. *Nature*, **511**, 583-586.

Howell J, Humphreys GS, Mitchell PB (2006) Changes in soil water repellence and its distribution in relation to surface microtopographic units after a low severity fire in eucalypt woodland, Sydney, Australia. *Australian Journal of Soil Research,* **44**, 205-217.

Hunt JE, Kelliher FM, Mcseveny TM, Ross DJ, Whitehead D (2004) Long-term carbon exchange in a sparse, seasonally dry tussock grassland. *Global Change Biology,* **10**, 1785-1800.

Jaksic V, Kiely G, Albertson J, Oren R, Katul G, Leahy P, Byrne KA (2006) Net ecosystem exchange of grassland in contrasting wet and dry years. *Agricultural and Forest Meteorology,* **139**, 323-334.

Jolly WM, Dobbertin M, Zimmermann NE, Reichstein M (2005) Divergent vegetation growth responses to the 2003 heat wave in the Swiss Alps. *Geophysical Research Letters,* **32**, doi:10.1029/2005GL023252.

Joos O, Hagedorn F, Heim A, Gilgen AK, Schmidt MWI, Siegwolf RTW, Buchmann N (2010) Summer drought reduces total and litter-derived soil CO_2_ effluxes in temperate grassland - clues from a ^13^C litter addition experiment. *Biogeosciences,* **7**, 1031-1041.

Kahmen A, Perner J, Buchmann N (2005) Diversity-dependent productivity in semi-natural grasslands following climate perturbations. *Functional Ecology,* **19**, 594-601.

Klein T, Yakir D, Buchmann N, Grünzweig JM (2014) Towards an advanced assessment of the hydrological vulnerability of forests to climate change-induced drought. *New Phytologist*, **201** (3), 712-716.

Klein Goldewijk K, Ramankutty N (2004) Land cover change over the last three centuries due to human activities: the availability of new global data sets. *Geojournal,* **61**, 335-344.

Knapp AK, Fay PA, Blair JM *et al.* (2002) Rainfall variability, carbon cycling, and plant species diversity in a mesic grassland. *Science,* **298**, 2202-2205.

Kreyling J, Wenigmann M, Beierkuhnlein C, Jentsch A (2008) Effects of extreme weather events on plant productivity and tissue die-back are modified by community composition. *Ecosystems,* **11**, 752-763.

Kurz WA, Dymond CC, Stinson G *et al.* (2008) Mountain pine beetle and forest carbon feedback to climate change. *Nature,* **452**, 987-990.

Lal R (2007) Carbon management in agricultural soils. *Mitigation and Adaptation Strategies for Global Change,* **12**, 303-322.

Larcher W (2003) *Physiological Plant Ecology,* Berlin, Springer.Lewis SL, Brando PM, Phillips OL, Van Der Heijden GMF, Nepstad D (2011) The 2010 Amazon drought. *Science,* **331**, 554.

Lindroth A, Lagergren F, Grelle A, Klemedtsson L, Langvall O, Weslien P, Tuulik J (2009) Storms can cause Europe-wide reduction in forest carbon sink. *Global Change Biology,* **15**, 346-355.

Lobell DB, Schlenker W, Costa-Roberts J (2011) Climate trends and global crop production since 1980. *Science,* **333**, 616-620.

Lobell DB, Sibley A, Ortiz-Monasterio JI (2012) Extreme heat effects on wheat senescence in India. *Nature Climate Change,* **2**, 1-4.

Loka DA, Oosterhuis DM (2010) Effect of high night temperatures on cotton respiration, ATP levels and carbohydrate content. *Environmental and Experimental Botany,* **68**, 258-263.

Ma SY, Baldocchi DD, Xu LK, Hehn T (2007) Inter-annual variability in carbon dioxide exchange of an oak/grass savanna and open grassland in California. *Agricultural and Forest Meteorology,* **147**, 157-171.

Mars Bulletin (2007) Forecast Update, 21 August. European Commission. Joint Research Centre, FU2007/05.

Martinez-Meier A, Sanchez L, Pastorino M, Gallo L, Rozenberg P (2008) What is hot in tree rings? The wood density of surviving Douglas-firs to the 2003 drought and heat wave. *Forest Ecology and Management,* **256**, 837-843.

McDowell NG, Beerling DJ, Breshears DD, Fisher RA, Raffa KF, Stitt M (2011) The interdependence of mechanisms underlying climate-driven vegetation mortality. *Trends in Ecology and Evolution,* **26**, 523-532.

McDowell NG, Ryan MG, Zeppel MJB, Tissue DT (2013) Improving our knowledge of drought-induced forest mortality through experiments, observations, and modeling. *New Phytologist,* **200**, 289-293.

Meir P, Woodward FI (2010) Amazonian rain forests and drought: response and vulnerability. *New Phytologist,* **187**, 553-557.

Morgan JA, LeCain DR, Pendall E *et al.* (2011) C_4_ grasses prosper as carbon dioxide eliminates desiccation in warmed semi-arid grassland. *Nature,* **476**, 202-205.

Negrón-Juárez R, Baker DB, Zeng H, Henkel TK, Chambers JQ (2010) Assessing hurricane-induced tree mortality in U.S. Gulf Coast forest ecosystems. *Journal of Geophysical Research - Biogeosciences,* **115**, G04030.

Nellemann C, MacDevette M, Manders T, Eickhout B, Svihus B, Prins AG, Kaltenborn BP (2009) *The Environmental Food Crisis. The Environment's Role in Averting Future Food Crises. A UNEP Rapid Response Assessment,* Birkeland Trykkeri AS, Norway, United Nations Environment Programme.

Nykänen M-L, Peltola H, Quine CP, Kellomäki S, Broadgate M (1997) Factors affecting snow damage of trees with particular reference to European conditions. *Silva Fennica,* **31**, 193-213.

Owensby CE, Ham JM, Knapp AK, Bremer D, Auen LM (1997) Water vapour fluxes and their impact under elevated CO_2_ in a C_4_-tallgrass prairie. *Global Change Biology,* **3**, 189-195.

Øygarden L (2003) Rill and gully development during an extreme winter runoff event in Norway. *Catena,* **50**, 217-242.

Page SE, Rieley JO, Banks CJ (2011) Global and regional importance of the tropical peatland carbon pool. *Global Change Biology,* **17**, 798-818.

Pan Y, Birdsey RA, Fang J *et al.* (2011) A large and persistent carbon sink in the world's forests. *Science,* **333**, 988-993.

Patterson DT (1995) Weeds in a changing climate. *Weed Science,* **43**, 685-701.

Pausas JG, Bradstock RA, Keith DA, Keeley JE (2004) Plant functional traits in relation to fire in crown-fire ecosystems. *Ecology,* **85**, 1085-1100.

Pausas JG, Keeley JE (2009) A burning story: the role of fire in the history of life. *Bioscience,* **59**, 593-601.

Peichl M, Leahy P, Kiely G (2011) Six-year stable annual uptake of carbon dioxide in intensively managed humid temperate grassland. *Ecosystems,* **14**, 112-126.

Peng SB, Huang JL, Sheehy JE *et al.* (2004) Rice yields decline with higher night temperature from global warming. *Proceedings of the National Academy of Sciences of the United States of America,* **101**, 9971-9975.

Phillips OL, Aragão LEOC, Lewis SL *et al.* (2009) Drought sensitivity of the Amazon rainforest. *Science,* **323**, 1344-1347.

Piao SL, Ciais P, Friedlingstein P *et al.* (2008) Net carbon dioxide losses of northern ecosystems in response to autumn warming. *Nature,* **451**, 49-52.

Pimentel D, Kounang N (1998) Ecology of soil erosion in ecosystems. *Ecosystems,* **1**, 416-426.

Porter JR, Semenov MA (2005) Crop responses to climatic variation. *Philosophical Transactions of the Royal Society B - Biological Sciences,* **360**, 2021-2035.

Ramankutty N, Evan AT, Monfreda C, Foley JA (2008) Farming the planet: 1. Geographic distribution of global agricultural lands in the year 2000. *Global Biogeochemical Cycles,* **22**, doi:10.1029/2007GB002952.

Rosati A, Metcalf SG, Buchner RP, Fulton AE, Lampinen BD (2006) Physiological effects of kaolin applications in well-irrigated and water-stressed walnut and almond trees. *Annals of botany*, **98**, 267-275.

Rouault G, Candau J-N, Lieutier F, Nageleisen L-M, Martin J-C, Warzee N (2006) Effects of drought and heat on forest insect populations in relation to the 2003 drought in Western Europe. *Annals of Forest Science,* **63**, 613-624.

Sala A, Piper F, Hoch G (2010) Physiological mechanisms of drought-induced tree mortality are far from being resolved. *New Phytologist,* **186**, 274-281.

Sánchez-Salguero R, Navarro-Cerrillo RM, Swetnam TW, Zavala MA (2012) Is drought the main decline factor at the rear edge of Europe? The case of southern Iberian pine plantations. *Forest Ecology and Management,* **271**, 158-169.

Schulze ED, Luyssaert S, Ciais P *et al.* (2009) Importance of methane and nitrous oxide for Europe's terrestrial greenhouse-gas balance. *Nature Geoscience,* **2**, 842-850.

Schwalm CR, Williams CA, Schaefer K *et al.* (2010) Assimilation exceeds respiration sensitivity to drought: A FLUXNET synthesis. *Global Change Biology,* **16**, 657-670.

Signarbieux C, Feller U (2011) Non-stomatal limitations of photosynthesis in grassland species under artificial drought in the field. *Environmental and Experimental Botany,* **71**, 192-197.

Singleton GR, Belmain SR, Brown PR, Aplin KP, Htwe NM (2010) Impacts of rodent outbreaks on food security in Asia. *Wildlife Research,* **37**, 355-359.

Skjemstad JO, Reicosky DC, Wilts AR, McGowan JA (2002) Charcoal carbon in U.S. agricultural soils. *Soil Science Society of America Journal,* **66**, 1249-1255.

Smith P (2004) Carbon sequestration in croplands: the potential in Europe and the global context. *European Journal of Agronomy,* **20**, 229-236.

Smith P (2012) Agricultural greenhouse gas mitigation potential globally, in Europe and in the UK: What have we learnt in the last 20 years? *Global Change Biology,* **18**, 35-43.

Smith P, Jones M, Osborne B, Wattenbach M (2010) The carbon and greenhouse gas budget of European croplands. *Agriculture, Ecosystems and Environment,* **139**, V-VI.

Sowerby A, Emmett BA, Tietema A, Beier C (2008) Contrasting effects of repeated summer drought on soil carbon efflux in hydric and mesic heathland soils. *Global Change Biology,* **14**, 2388-2404.

Sukhinin AI, French NHF, Kasischke ES *et al.* (2004) AVHRR-based mapping of fires in Russia: New products for fire management and carbon cycle studies. *Remote Sensing of Environment,* **93**, 546-564.

Sutherland JP, Baharally V (2003) The influence of weather on the population dynamics of rice stink bug and the implications for integrated pest management. *International Journal of Pest Management,* **49**, 335-342.

Talmon Y, Sternberg M, Grünzweig JM (2011) Impact of rainfall manipulations and biotic controls on soil respiration in Mediterranean and desert ecosystems along an aridity gradient. *Global Change Biology,* **17**, 1108-1118.

Tarnocai C, Canadell JG, Schuur EaG, Kuhry P, Mazhitova G, Zimov S (2009) Soil organic carbon pools in the northern circumpolar permafrost region. *Global Biogeochemical Cycles,* **23**, GB2023, doi:2010.1029/2008GB003327.

Thomey ML, Collins SL, Vargas R, Johnson JE, Brown RF, Natvig DO, Friggens MT (2011) Effect of precipitation variability on net primary production and soil respiration in a Chihuahuan Desert grassland. *Global Change Biology,* **17**, 1505-1515.

Thothong W, Huon S, Janeau JL *et al.* (2011) Impact of land use change and rainfall on sediment and carbon accumulation in a water reservoir of North Thailand. *Agriculture, Ecosystems and Environment,* **140**, 521-533.

Tian H, Melillo JM, Kicklighter DW, McGiure AD, Helfrich III J, Moore III B, Vörösmarty CJ (1998) Effect of interannual climate variability on carbon storage in Amazonian ecosystems. *Nature,* **396**, 664-667.

Turetsky M, Wieder K, Halsey L, Vitt D (2002) Current disturbance and the diminishing peatland carbon sink. *Geophysical Research Letters,* **29**, 1526, doi:1510.1029/2001GL014000.

Turetsky MR, Donahue WF, Benscoter BW (2011a) Experimental drying intensifies burning and carbon losses in a northern peatland. *Nature Communications,* **2**, 514.

Turetsky MR, Kane ES, Harden JW, Ottmar RD, Manies KL, Hoy E, Kasischke ES (2011b) Recent acceleration of biomass burning and carbon losses in Alaskan forests and peatlands. *Nature Geoscience,* **4**, 27-31.

Valinger E, Fridman J (1999) Models to assess the risk of snow and wind damage in pine, spruce, and birch forests in Sweden. *Environmental Management,* **24**, 209-217.

van der Molen MK, Dolman AJ, Ciais P *et al.* (2011) Drought and ecosystem carbon cycling. *Agricultural and Forest Meteorology,* **151**, 765-773.

van der Velde M, Tubiello FN, Vrieling A, Bouraoui F (2012) Impacts of extreme weather on wheat and maize in France: evaluating regional crop simulations against observed data. *Climatic Change,* **113**, 751-765.

van der Velde M, Wriedt G, Bouraoui F (2010) Estimating irrigation use and effects on maize yield during the 2003 heatwave in France. *Agriculture, Ecosystems amd Environment,* **135**, 90-97.

van der Werf GR, Dempewolf J, Trigg SN *et al.* (2008) Climate regulation of fire emissions and deforestation in equatorial Asia. *Proceedings of the National Academy of Sciences of the United States of America,* **105**, 20350-20355.

van der Werf GR, Randerson JT, Giglio L *et al.* (2010) Global fire emissions and the contribution of deforestation, savanna, forest, agricultural, and peat fires (1997-2009). *Atmospheric Chemistry and Physics,* **10**, 11707-11735.

van Oost K, Quine TA, Govers G *et al.* (2007) The impact of agricultural soil erosion on the global carbon cycle. *Science,* **318**, 626-629.

Vargas R, Collins SL, Thomey ML, Johnson JE, Brown RF, Natvig DO, Friggens MT (2012) Precipitation variability and fire influence the temporal dynamics of soil CO_2_ efflux in an arid grassland. *Global Change Biology,* **18**, 1401-1411.

Vivchar A (2011) Wildfires in Russia in 2000-2008: estimates of burnt areas using the satellite MODIS MCD45 data. *Remote Sensing Letters,* **2**, 81-90.

Xu L, Baldocchi DD, Tang J (2004) How soil moisture, rain pulses, and growth alter the response of ecosystem respiration to temperature. *Global Biogeochemical Cycles,* **18**, GB4002, doi:4010.1029/2004GB002281.

Zavalloni C, Gielen B, Lemmens CMHM *et al.* (2008) Does a warmer climate with frequent mild water shortages protect grassland communities against a prolonged drought? *Plant and Soil,* **308**, 119-130.
